# Classical swine fever virus non-structural protein 5B hijacks host METTL14-mediated m^6^A modification to counteract host antiviral immune response

**DOI:** 10.1371/journal.ppat.1012130

**Published:** 2024-03-29

**Authors:** Jing Chen, Hui-xin Song, Jia-huan Hu, Ji-shan Bai, Xiao-han Li, Rui-cong Sun, Bing-qian Zhao, Mei-zhen Li, Bin Zhou

**Affiliations:** 1 MOE Joint International Research Laboratory of Animal Health and Food Safety, College of Veterinary Medicine, Nanjing Agricultural University, Nanjing, China; 2 Guizhou Provincial Center for Disease Control and Prevention, Guiyang, China; Thomas Jefferson University - Center City Campus: Thomas Jefferson University, UNITED STATES

## Abstract

Classical Swine Fever (CSF), caused by the Classical Swine Fever Virus (CSFV), inflicts significant economic losses on the global pig industry. A key factor in the challenge of eradicating this virus is its ability to evade the host’s innate immune response, leading to persistent infections. In our study, we elucidate the molecular mechanism through which CSFV exploits m^6^A modifications to circumvent host immune surveillance, thus facilitating its proliferation. We initially discovered that m^6^A modifications were elevated both *in vivo* and *in vitro* upon CSFV infection, particularly noting an increase in the expression of the methyltransferase METTL14. CSFV non-structural protein 5B was found to hijack HRD1, the E3 ubiquitin ligase for METTL14, preventing METTL14 degradation. MeRIP-seq analysis further revealed that METTL14 specifically targeted and methylated TLRs, notably TLR4. METTL14-mediated regulation of TLR4 degradation, facilitated by YTHDF2, led to the accelerated mRNA decay of TLR4. Consequently, TLR4-mediated NF-κB signaling, a crucial component of the innate immune response, is suppressed by CSFV. Collectively, these data effectively highlight the viral evasion tactics, shedding light on potential antiviral strategies targeting METTL14 to curb CSFV infection.

## Author summary

A significant concern arises from the enduring infection attributable to the suppression of the host innate immune response subsequent to CSFV infection. Our recent studies have excitingly unveiled the molecular mechanisms by which CSFV manipulates host methylation modifications to evade innate immune surveillance, thereby enhancing viral proliferation. Notably, CSFV non-structural protein 5B strategically commandeered the host factor HRD1. This interaction prevented HRD1 from ubiquitinating METTL14, leading to increased intracellular stability and expression of METTL14. Significantly, MeRIP-seq analysis revealed that METTL14 specifically targeted TLR4, recruiting YTFDH2 to facilitate TLR4 degradation, which in turn inhibited the TLR4 regulated NF-κB pathway. This suppression of the innate immune response resulted in a marked increase in CSFV replication. This distinct regulatory mechanism, unique to CSFV within the Pestivirus genus, illuminates the strategy of virus in exploiting m^6^A modification to bypass innate immunity, specifically focusing on the suppression of the NF-κB pathway at its source.

## Introduction

Classical Swine Fever (CSF), caused by the Classical Swine Fever Virus (CSFV), is clinically characterized by high fever, hemorrhages in the skin and viscera, as well as respiratory and gastrointestinal syndromes, leading to substantial economic losses in the global pig industry [1,2]. The genus Pestivirus, within the *Flaviviridae* family, is recognized to include four species: Bovine Viral Diarrhea Virus 1(BVDV-1), Bovine Viral Diarrhea Virus 2 (BVDV-2), Classical Swine Fever Virus (CSFV), and Border Disease Virus (BDV), all known for causing diseases in livestock [3,4]. The genome of CSFV is a single-stranded, positive-sense RNA, approximately 12.3kb in length, encoding a polyprotein that includes 5’-UTR, an open reading frame (ORF), and 3’-UTR. This polyprotein can be further cleaved into four structural proteins (capsid protein C, envelope glycoproteins Erns, E1, and E2) and eight non-structural proteins (Npro, p7, NS2, NS3, NS4A, NS4B, NS5A, and NS5B) [5]. CSFV typically enters pigs through oral and nasal routes, rapidly disseminating to lymphoid tissues before spreading to various parenchymal organs, accompanied by the induction of immune suppression. The detection of immunosuppression often precedes seroconversion and the appearance of clinical symptoms, underscoring its importance in early diagnosis and research into viral pathogenesis [6–9].

Previous researches within the *Flaviviridae* family have revealed significant alterations in intracellular gene expression and post-transcriptional modifications following viral infection, indicating a complex interplay between viral infection and the modulation of host gene expression. The host counters viral infection by adjusting the expressions of relevant genes, whereas the virus manipulates host transcription to circumvent defense mechanisms and enhance its replication. Therefore, the variable expression levels of antiviral host factors play a crucial role in determining the outcome of viral infections in the *Flaviviridae* family [10–14].

The expression of host genes during viral infections in the *Flaviviridae* family can be regulated through RNA post-transcriptional modifications, which include chemical modifications of RNA [15, 16]. Currently, the most widespread internal modification in mRNA is m^6^A, governed by specific cellular proteins [17]. The "writers" complex, comprising METTL3-METTL14-WTAP, targets the DRA*CH motif in mRNA sequences (where D = G/A/U, R = G/A, H = U/A/C, * denotes a modified A), facilitating the methylation of adenosine residues in mRNAs [18,19]. RNA binding protein "readers" recognize m^6^A modification sites, thereby regulating mRNA metabolism and affecting processes such as mRNA splicing, nuclear export, stability, translation, and structure [20]. For instance, upon Vesicular Stomatitis Virus (VSV) infection in mouse peritoneal macrophages, a significant increase in m^6^A levels was observed between 8 to 12 hours post-infection (hpi), followed by a reduction at 24 hpi, mirroring the initial spike and subsequent reduction in viral load [21]. Recent studies have shown that infections by Human Enterovirus 71 (EV71) and Human Cytomegalovirus (HCMV) lead to a notable upsurge in the expression of METTL3, METTL14, YTHDF1-3, and YTHDC1 in host cells, without affecting the expression of "eraser" proteins [22,23]. Additionally, the Epstein-Barr virus (EBV) latent antigen EBNA3C has been found to increase the expression of METTL14 by approximately 2.5-fold [24]. These findings imply that viral infection primarily affects the protein expression of "writers" and "readers", subsequently influencing host m^6^A modification.

Toll-like receptors (TLRs) serve as fundamental sensor molecules within the innate immune system. They possess the ability to detect conserved viral structures, thereby triggering innate immune responses [25]. TLR3, TLR7, TLR8, and TLR9 play the key roles in detecting diverse forms of viral nucleic acids—such as double-stranded RNA (dsRNA), single-stranded RNA (ssRNA), and CpG DNA—thus initiating host antiviral immune responses. Additionally, TLR2 and TLR4 are implicated in recognizing specific viral envelope proteins or components, facilitating the production of intracellular inflammatory cytokines [26–28]. Current reports on CSFV-induced innate immunity response focus primarily on the activation of key immune cells such as macrophages and plasma-like dendritic cells, alongside studying on the release of crucial factors such as IFN-I and various pro-inflammatory cytokines, notably IL-6, IL-10, IL-12, and TNF-α [29–32]. Moreover, CSFV infection has been identified to inhibit the nuclear factor kappa B (NF-κB) signaling pathway both *in vitro* and *in vivo*, but the molecular mechanisms of inhibition have not been elucidated [32]. Furthermore, CSFV non-structural protein 4B (NS4B) has been found to stimulate the production of downstream genes related to TLR7. These findings imply that CSFV non-structural proteins potentially play a crucial role in mediating innate immune response via TLRs [33].

CSFV, a significant member of the *Flaviviridae* family, uniquely infects domestic pigs and wild boars. A notable aspect of CSFV is its capacity to establish persistent infection by evading host immune surveillance, which poses a major challenge in eradicating the virus from the host. While previous studies have acknowledged that methylation modification plays a role in regulating innate immunity during viral infection, specific investigations on influence of CSFV on this process have been limited. In our study, we focused on elucidating the pivotal role of METTL14 in modulating the TLR4 and the downstream NF-κB signaling pathway, which were integral to the pathogenesis of CSFV infection. Our findings demonstrate that the NS5B protein of CSFV engages in an interaction with HRD1, thereby inhibiting the ubiquitination of METTL14. This interaction leads to an increase in the endogenous expression levels of METTL14, which in turn results in augmented m^6^A modifications both *in vitro* and *in vivo*. Concurrently, METTL14 specifically targets the 3’UTR region of TLR4 mRNA, orchestrating its degradation through YTHDF2- mediated pathways. This process leads to a reduction in the protein expression of TLR4, a molecule known to activate the NF-κB pathway and stimulate the production of associated inflammatory mediators cytokines. By attenuating TLR4 protein synthesis at its origin, METTL14 exerts a suppressive effect on the NF-κB signaling pathway, thereby facilitating an environment conducive to the replication of CSFV.

## Result

### CSFV infection enhances the host m^6^A modification

**Fig 1:**
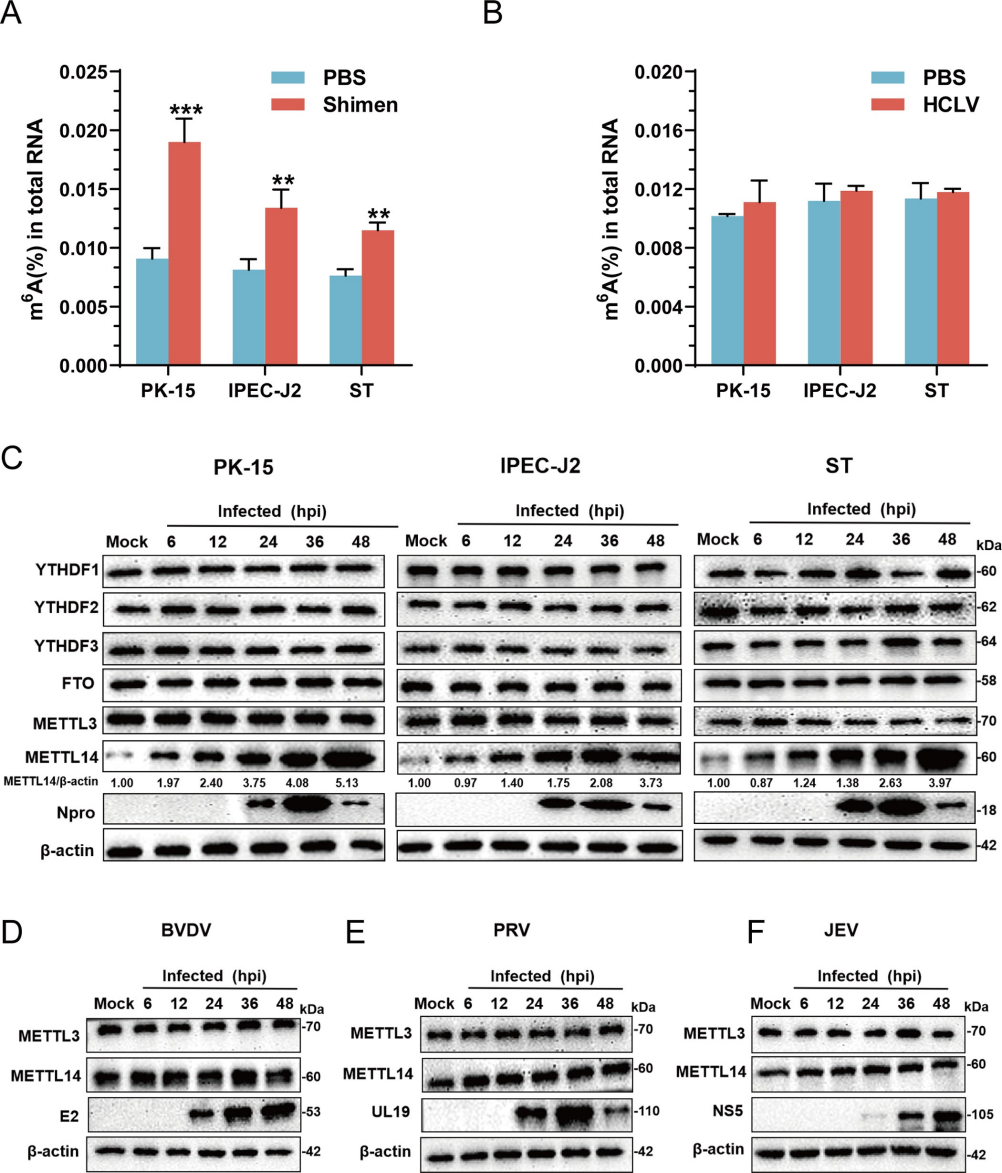
m^6^A and METTL14 levels increase upon CSFV infection. (A and B) PK-15, IPEC-J2, and ST cells were infected with CSFV (Shimen and HCLV strain) (MOI = 1) and the global m^6^A levels were measured by EpiQuik m^6^A RNA methylation quantification kit. (C) PK-15, IPEC-J2, and ST cells were infected with CSFV (MOI = 1). At 6, 12, 24, 36, and 48 hpi, cells were harvested and then subjected to western blotting using the indicated antibodies against YTHDF1, YTHDF2, YTHDF3, FTO, METTL3, METTL14, Npro and β-actin. (D-F) MDBK cells was infected with BVDV (MOI = 1) and PK-15 cells were infected with PRV (MOI = 0.1) or JEV (MOI = 0.5). At 6, 12, 24, 36, and 48 hpi, cells were harvested and then subjected to western blotting by using the indicated antibodies as follows: rabbit anti- METTL3/METTL14 antibody, rabbit anti-Npro antibody, mouse anti-E2/UL19/ NS5 antibody and rabbit anti-β-actin antibody. These data are presented as the mean ± SD of data from three independent experiments. **p < 0.01, ***p < 0.001.

In this context, we conducted a study to ascertain if CSFV infection altered host m^6^A RNA methylation. Therefore, to investigate whether CSFV infection alters the host m^6^A modification, PK-15, IPEC-J2, and ST cells were infected with CSFV (the Shimen and HCLV strains), harvested and lysed after 48 hpi. The EpiQuik m^6^A RNA Methylation Quantification Kit was utilized to measure the global m^6^A modification levels in the mRNAs of three cells. The results revealed a significant upregulation in m^6^A modification levels in cells infected with the Shimen strain, whereas no notable changes were observed in cells infected with the HCLV strain (Fig 1A and 1B). This suggests that the virulent strain of CSFV induces an increase in m^6^A methylation, a feature not observed with the vaccine strain. Moreover, to identify the specific m^6^A modification- associated proteins that may regulate CSFV replication, the PK-15, IPEC-J2, and ST cells were infected with CSFV (MOI = 1) and samples were harvested at 6, 12, 24, 36, and 48 hpi for western blotting. This results indicated that CSFV infection increasesd the protein expression of METTL14 in three cells, while the expression of other proteins such as YTHDF1, YTHDF2, YTHDF3, FTO, and METTL3 remained unchanged (Fig 1C). To ascertain whether the increase in METTL14 expression was specific to CSFV infection, MDBK cells were infected with BVDV, while PK-15 cells were separately infected with Pseudorabies Virus (PRV) and Japanese Encephalitis Virus (JEV). These cells were harvested and lysed at 6, 12, 24, 36, and 48 hpi for western blotting. The results showed that infections with BVDV, PRV, and JEV did not alter the protein expression of METTL3 or METTL14 (Fig 1D, 1E and 1F), indicating that the upregulation of METTL14 protein expression is a specific response to CSFV infection. Together, these data indicate that viral infection induces the upregulation of METTL14 protein-mediated methylation in host cells.

**Fig 2:**
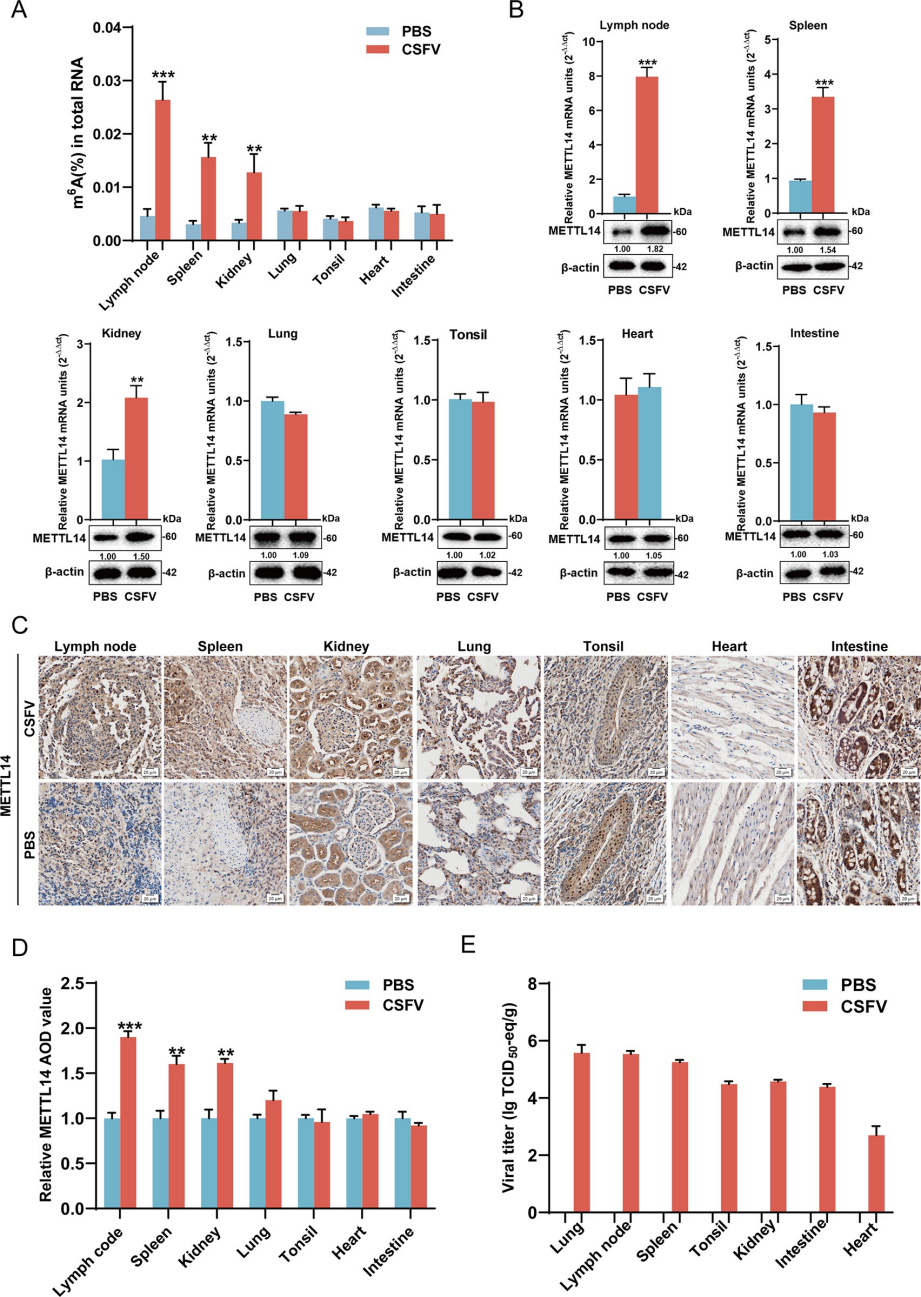
m^6^A and METTL14 levels increase *in vivo*. (A) Lymph node, spleen, kidney, lung, tonsil, heart, and intestine of pigs infected with or without CSFV (10^5^ TCID_50_) were extracted RNA and tested the global m^6^A levels using EpiQuik m^6^A RNA methylation quantification kit. (B) Lymph node, spleen, kidney, lung, tonsil, heart, and intestine from CSFV-infected or PBS-treated pigs were extracted RNA or lysed for RT-qPCR or western blotting to measure the METTL14 mRNA and protein levels. (C and D) METTL14 levels in lymph node, spleen, kidney, lung, tonsil, heart, and intestine from CSFV-infected or PBS-treated pigs were measured using IHC assay. 16×magnification (scale bar, 20 μm). (E) The viral titer in the seven tissues from CSFV-infected or PBS-treated pigs were measured using RT-qPCR. These data are presented as the mean ± SD of data from three independent experiments. **p < 0.01, ***p < 0.001.

Curiously, to confirm whether viral infection *in vivo* also caused the changes of m^6^A modification levels, the pigs were separated into two groups: one group (n = 5) infected with CSFV (10^5^ TCID_50_) as the infected group, another group (n = 5) treated with PBS as the uninfected mock. At 9 days post challenge (dpc), infected pigs with the typical clinical symptoms such as high fever, diarrhea, and epidermal bleeding were sacrificed. The m^6^A modification levels of multiple organs of the two groups were measured using the EpiQuik m^6^A RNA methylation quantification kit. As shown in Fig 2A, the m^6^A methylation levels of lymph node, spleen, and kidney in Shimen-infected group significantly increased approximately 5.75, 5.11, and 3.84-fold higher than that in uninfected group. However, there were no significant changes of the m^6^A modification levels in the lung, tonsil, heart, and intestine. Next, mRNA synthesis and protein expression of METTL3 and METTL14 in infected tissue samples were detected using RT-qPCR and western blotting. The results showed that the mRNA and protein expressions of METTL14 in lymph node, spleen, and kidney increased upon viral infection, while there were no significant changes in the lung, tonsil, heart, and intestine. We also found that there were no significant changes in mRNA synthesis and protein expressions of METTL3 in these infected tissues (Figs 2B and S1A). Furthermore, the protein expression of METTL3 and METTL14 in infected and uninfected tissues were demonstrated using the IHC assay. The results showed that the protein expressions of METTL14 in infected lymph node, spleen, and kidney in Shimen-infected group significantly increased approximately 1.90-, 1.60-, and 1.61-fold higher than that in uninfected group. Conversely, no significant differences of protein expressions of METTL14 were detected in the lung, tonsil, heart, and intestine between the two groups (Fig 2C and 2D). Regardless of CSFV infection or not, there were no significant differences of protein expressions of METTL3 among all the tissues between the two groups (S1B and S1C Fig). We also used RT-qPCR to detect viral titers in all the tissues. As shown in Fig 2E, viral titers in the infected group ranging from 3.54 to 5.98 lgTCID_50_ eq/g. Notably, higher virus titers were detected in the lung, lymph node, spleen, and tonsil, reaching 5.98, 5.63, 5.10, and 5.00 lgTCID_50_ eq/g. Viral titers in the kidney and intestine were 4.95 and 4.42 lgTCID_50_ eq/g, while that in the heart was the lowest at 3.54 lgTCID_50_ eq/g, and no viral titers were detected in the group treated with PBS. Overall, these results indicated that CSFV infection *in vivo* resulted in an increase in METTL14 expressions in lymph node, spleen and kidney, followed by an increase in m^6^A modification levels in infected pigs. Interestingly, the levels of METTL14 expression and m^6^A modification were not changed in the lung and tonsil with high viral titers. We speculated that CSFV entered the lungs following oral and nasal pathway, leading to acute infection. Consequently, it is conceivable that the delay observed in the m^6^A modification reaction might be attributed to these acute stages of infection.

### METTL14 is essential for CSFV replication

**Fig 3:**
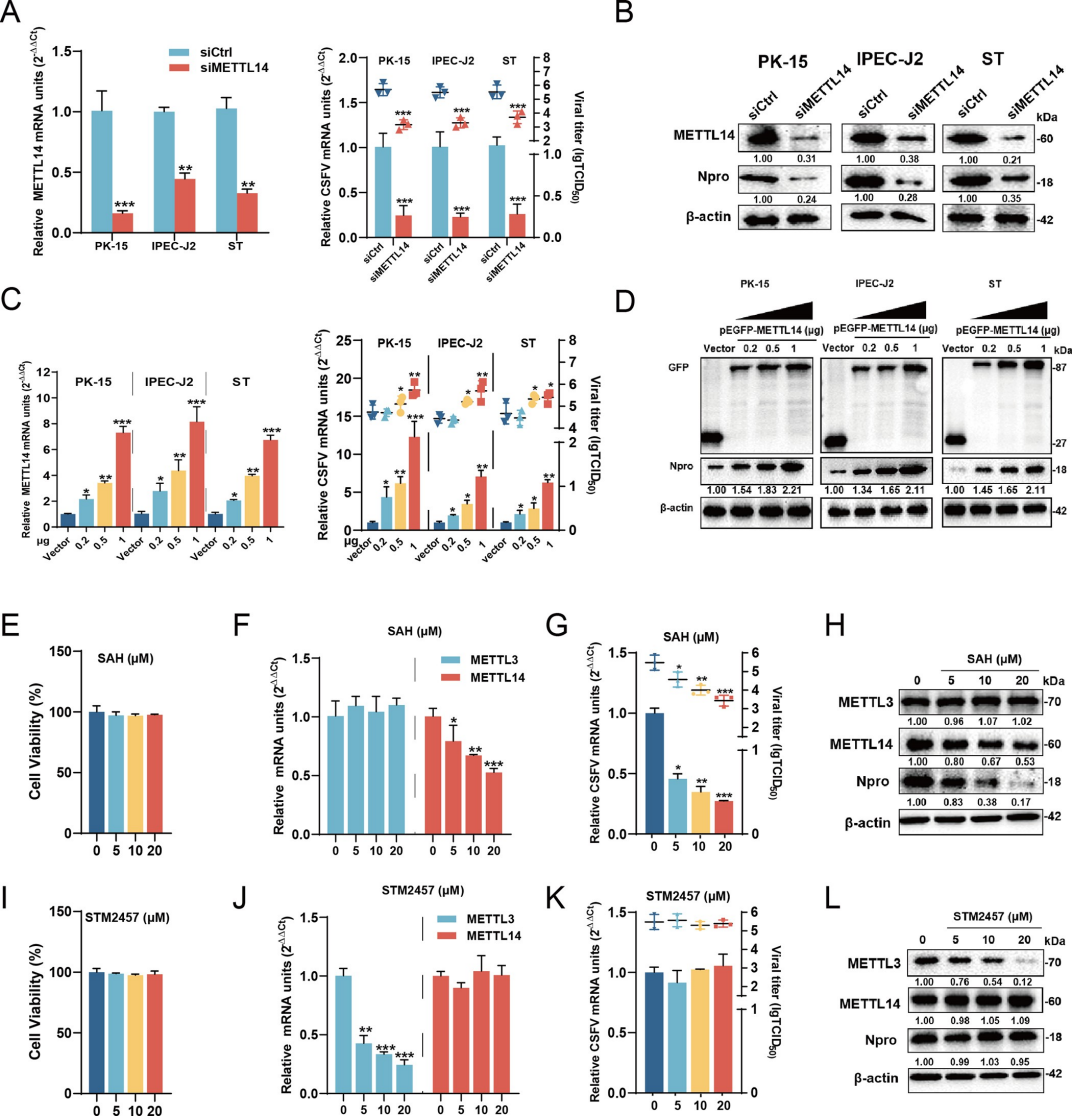
METTL14 promotes CSFV replication. (A and C) PK-15, IPEC-J2, and ST cells were transfected with siMETTL14 or siCtrl (A) or pEGFP-METTL14 (0.2, 0.5, and 1 μg) (C) and infected with CSFV (MOI = 1). At 24 hpi, cells were extracted RNA or lysated for RT-qPCR or virus titration. (B and D) PK-15, IPEC-J2, and ST cells were transfected with siMETTL14 or siCtrl (B) or pEGFP-METTL14 (0.2, 0.5, and 1 μg) (D) and infected with CSFV (MOI = 1). At 24 hpi, cells were harvested for western blotting using the indicated antibodies against GFP, METTL14, Npro, and β-actin antibody. (E and I) Cell viability upon SAH and STM2457 were assessed using the CCK-8 assay kit. (F, G, J, and K) PK-15 cells were treated with SAH (F and G) or STM2457 (5, 10 and 20 μM) (J and K) and infected with CSFV (MOI = 1). At 24 hpi, cells were extracted RNA or lysated and subjected to RT-qPCR or virus titration. These data are presented as the mean ± SD of data from three independent experiments. (H and L) PK-15 cells were treated with SAH (H) or STM2457 (5, 10 and 20 μM) (L) and infected with CSFV (MOI = 1). At 24 hpi, cells were harvested and subjected to western blotting using the indicated antibodies against METTL3, METTL14, Npro, and β-actin. These data are presented as the mean ± SD of data from three independent experiments. *p < 0.05, **p < 0.01, ***p < 0.001.

Building on the evidence that CSFV infection increases METTL14 expression both *in vivo* and *in vitro*, we hypothesized that METTL14 played a crucial role in the life cycle of CSFV. To test this hypothesis, PK-15, IPEC-J2, and ST cells were transfected with siRNAs targeting METTL14 or METTL3 for 24 h, and infected with CSFV (MOI = 1). At 24 hpi, cells were harvested and subjected to RT-qPCR, virus titration_,_ and western blotting. The results indicated a significant reduction in viral RNA synthesis, virus titers and Npro protein expression in the three METTL14-knockdown cells. As shown in Fig 3A and 3B, there were a respective decrease in the viral titers by 2.53, 2.20, and 1.83 lgTCID_50_ upon the knockdown of METTL14. Conversely, compared to the cells treated with siCtrl, knockdown of METTL3 did not affect the viral RNA generation, virus titers and Npro protein expression (S2A and S2B Fig). Simultaneously, three cells transfected with the constructs pEGFP-METTL3 or -METTL14 of different concentrations were infected with CSFV (MOI = 1), harvested and lysed for RT-qPCR, virus titration and western blotting at 24 hpi. The overexpression of METTL14 significantly enhanced viral RNA synthesis, virus titers and Npro protein expression in three cells. As shown in Fig 3C and 3D, no changes in virus titers were observed in cells transfected with 0.2 μg of pEGFP- METTL14. Notably, upon transfection with 0.5 μg of the constructs, viral titers increased by 0.56, 0.46, and 0.47 lgTCID_50,_ respectively. Furthermore, transfection with 1 μg of pEGFP-METTL14 resulted in an elevation of viral titers by 1.10, 1.00, and 0.73 lgTCID_50_, respectively. These data indicated a dose-dependent upregulation of viral replication in these cell lines. However, overexpression of METTL3 did not influence virus production (S2C and S2D Fig). To further validate the roles of METTL3 and METTL14 in CSFV replication, we used two drugs SAH as an inhibitor of the METTL3-METTL14 heterodimer complex (METTL3-14) and STM2457 as an effective, selective, and orally active inhibitor targeting METTL3. To ensure the proper progression of subsequent experiments, cell viability was evaluated following exposure to each drug using the Cell Counting Kit-8. As shown in Fig 3E and 3I, the concentrations of SAH and STM2457 utilized in this study were nontoxic. PK-15 cells treated with different concentrations of SAH or STM2457 (5, 10, and 20 μM) for 2 h prior to CSFV infection (MOI = 1) were collected and lysed for RT-qPCR, virus titration and western blotting at 24 hpi. The results demonstrated that SAH significantly reduced mRNA synthesis and METTL14 protein expression, without noticeably affecting METTL3, leading to an inhibition of viral replication (Fig 3F, 3G and 3H). Conversely, STM2457 exhibited significant inhibition in the mRNA and protein levels of METTL3 but had no effect on that of METTL14. As a result, its influence did not extend to viral mRNA generation, virus titer or Npro protein expression (Fig 3J, 3K and 3L). In conclusion, our data suggest that CSFV replication is dependent on METTL14, rather than METTL3, highlighting the specific role of METTL14 in the viral life cycle.

### CSFV NS5B is responsible for METTL14 upregulation

**Fig 4:**
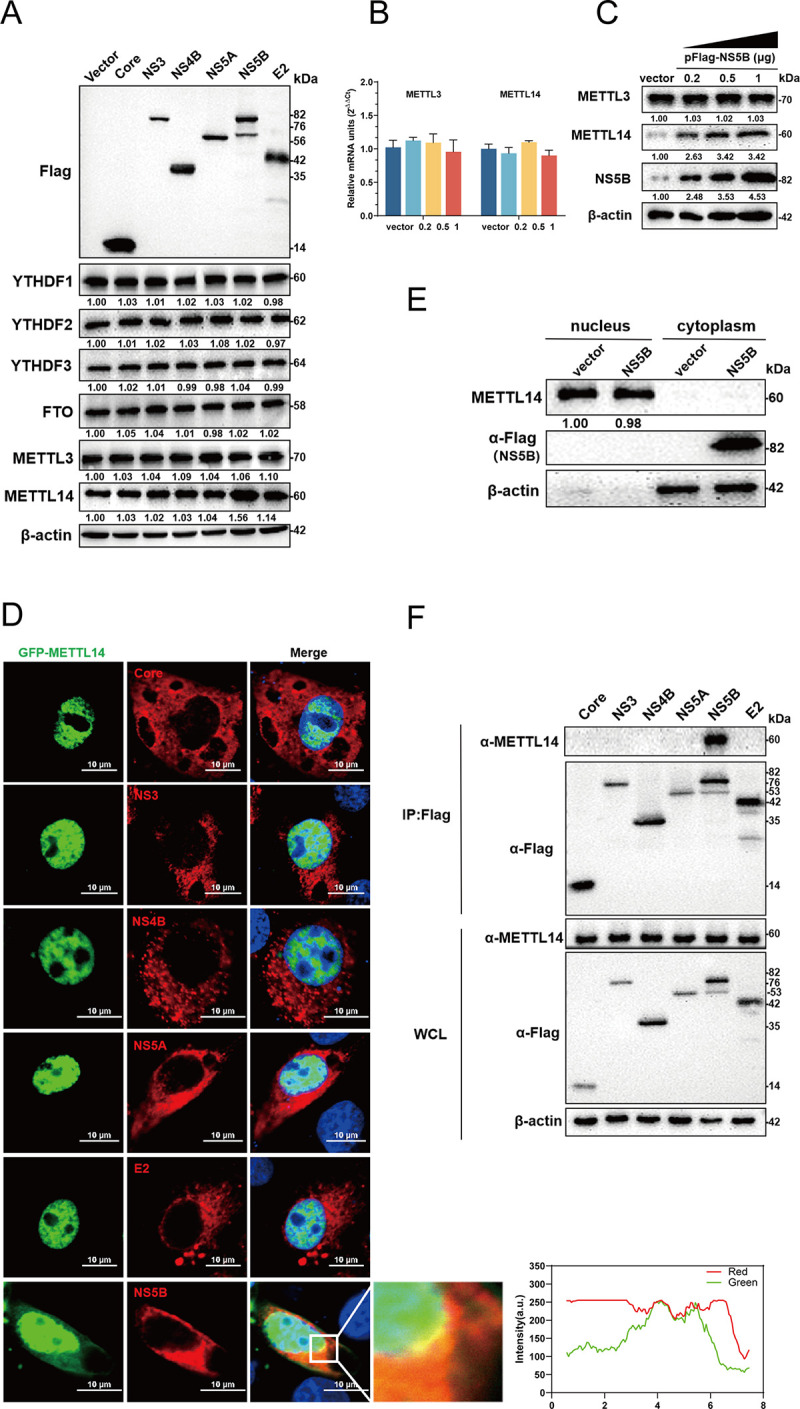
CSFV NS5B interacts with METTL14 around the nucleus. (A) PK-15 cells were transfected with indicated plasmids (pFlag-Core,-NS3, -NS4B,-NS5A,-NS5B, or -E2) or vector for 48 hpt, then harvested and subjected to western blotting using the indicated antibodies against Flag, YTHDF1, YTHDF2, YTHDF3, FTO, METTL3, METTL14, and β-actin. (B and C) PK-15 cells were transfected with pFlag-NS5B (0.2, 0.5, and 1 μg). At 24 hpi, cells were harvested and subjected to RT-qPCR and western blotting, respectively, as described above. (D) PK-15 cells were transfected with indicated plasmids (pFlag-Core, -NS3, -NS4B, -NS5A,-NS5B, or -E2), then shifted to 37°C for 24 h. Cells were fixed and subjected to immunofluorescent by using mouse anti-Flag antibody. The nuclei were stained with DAPI. Bars = 10 μm. (E) PK-15 cells transfected with pFlag-NS5B or vector were lysated and subjected to nucleoplasmic separation assay. (F) HEK-293T cells were transfected with indicated plasmids (pFlag-Core, -NS3, -NS4B, -NS5A, -NS5B, or -E2) and pEGFP-METTL14, then harvested for immunoprecipitation by using mouse anti-Flag antibody and whole-cell lysates were subjected to western blotting by using rabbit anti-METTL14 antibody or mouse anti-Flag antibody, along with β-actin as a loading control. These data are presented as the mean ± SD of data from three independent experiments. *p < 0.05, **p < 0.01, ***p < 0.001.

Given the observed upregulation of METTL14 protein expression following CSFV infection and its subsequent enhancement of CSFV replication, we hypothesized that viral proteins might be involved in the regulation of METTL14. To investigate this, PK-15 cells were transfected with constructs of various CSFV proteins, including pFlag-Core, -NS3, -NS4B, -NS5A, -NS5B, and -E2 for 24 h, and harvested and lysed for western blotting. The results indicated that among these key viral proteins, only the overexpression of NS5B led to an increase in the endogenous expression of METTL14 protein (Fig 4A). Notably, the overexpression of NS5B did not influence the expression levels of other five m^6^A modification-related proteins. Further, to assess the impact of different doses of NS5B on METTL3 and METTL14, cells transfected with pFlag-NS5B at various concentrations (0.2, 0.5, and 1 μg) were also harvested and analyzed. RT-qPCR result demonstrated that overexpression of NS5B did not affect the mRNA levels of METTL3 and METTL14. However, western blotting result revealed a dose-dependent increase in METTL14 protein expression, while METTL3 protein expression remained unaffected (Fig 4B and 4C). To explore the potential interaction between NS5B and METTL14, constructs pFlag-Core, -NS3, -NS4B, -NS5A, -NS5B, or -E2 and pEGFP- METTL14 were co-transfected into HEK-293T or PK-15 cells for Co- immunoprecipitation (Co-IP) and confocal fluorescence microscopy assays. The results, as shown in Fig 4D and 4F, showed that METTL14 co-localized with NS5B near the nucleus, but not with other viral proteins (Core, NS3, NS4B, NS5A, or E2). The Co-IP results corroborated the findings from the confocal fluorescence microscopy, with only NS5B being observed to coprecipitate with METTL14. Cells transfected with pFLAG-NS5B or vector were lysated and subjected to nucleoplasmic separation assay. However, following the overexpression of NS5B, there was no observed change in the distribution of endogenous METTL14 within the cytoplasm and nucleus (Fig 4E), suggesting that NS5B did not influence the intracellular localization of METTL14. Taken together, these results substantiate the interaction between NS5B and METTL14, underscoring the specific role of NS5B in regulating METTL14 protein expression, which in turn affects CSFV replication.

### CSFV NS5B interacts with HRD1 for protecting METTL14 from degradation

**Fig 5:**
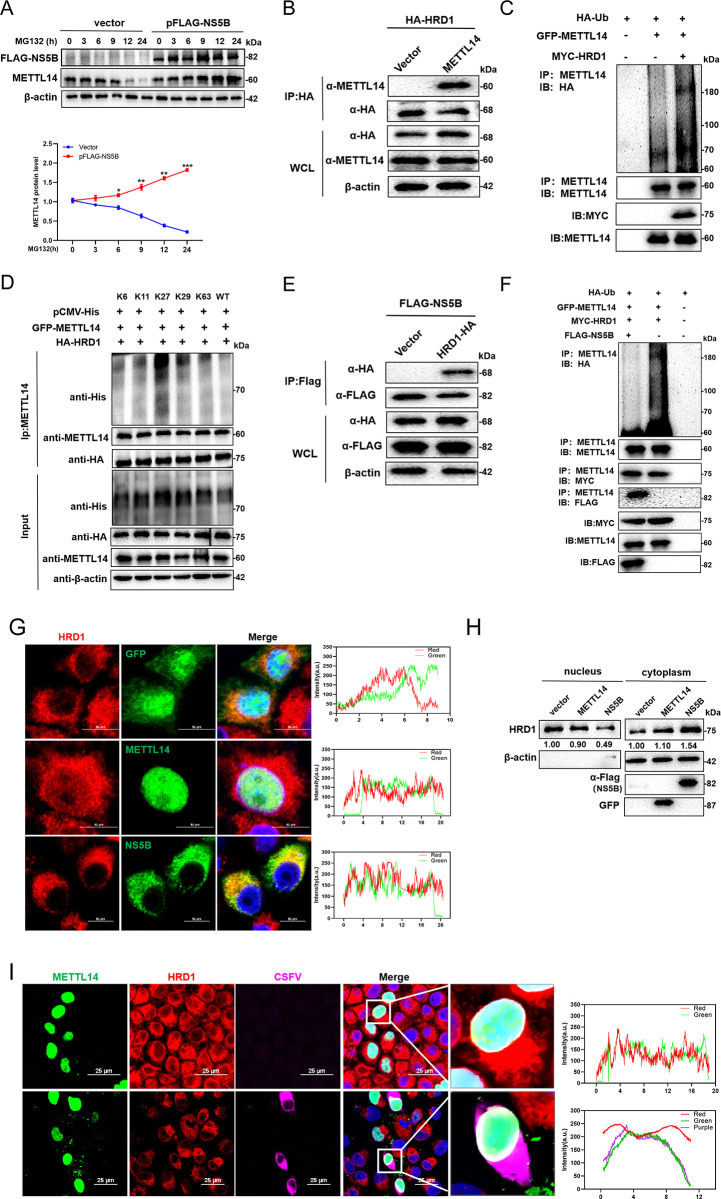
CSFV NS5B interacts with HRD1 and inhibits ubiquitination of METTL14. (A) PK-15 cells transfected with pFlag-NS5B were treated with MG132 (10 μM) for indicated times and then harvested for western blotting by using rabbit anti-METTL14 antibody, mouse anti-Flag antibody, and β-actin as a loading control. (B and E) HEK-293T cells were co-tansfected with pHA-HRD1 and pEGFP-METTL14 or pHA-HRD1 and pFlag-NS5B and then harvested for Co-IP by using rabbit anti-HA (B) or mouse anti-Flag (E) antibody. The whole-cell lysates were subjected to western blotting by using rabbit anti-HA/METTL14 antibody or mouse anti-Flag antibody, along with β-actin as a loading control. These data are representative of three independent experiments. (C, D and F) HEK-293T cells were transfected with pEGFP-METTL14, pMYC-HRD1, and pHA-ubiquitin (C) or pEGFP-METTL14, pHA-HRD1, and pCMV-His-ubiquitin (wild-type or -K6, -K11, -K27, -K29, or -K63) (D) or pFlag-NS5B, pMYC-HRD1, pHA-ubiquitin and pEGFP-METTL14 (F) and then harvested for Co-IP by using rabbit anti- METTL14 antibody. The whole-cell lysates were subjected to western blotting by using rabbit anti-HA/METTL14/MYC/His antibody or mouse anti-Flag antibody, along with β-actin as a loading control. (G) PK-15 cells transfected with pEGFP-C1, pEGFP-METTL14, or pFlag-NS5B for 24 h were fixed and subjected to immunofluorescent by using rabbit anti-HRD1 (red) antibody and mouse anti-Flag antibody (green). The nuclei were stained with DAPI. Bars = 10 μm. (H) PK-15 cells transfected with pEGFP-METTL14, pFlag-NS5B or vector were lysated and subjected to nucleoplasmic separation assay. (I) PK-15 cells transfected with pEGFP-METTL14 were infected with CSFV or not (MOI = 1). At 24 hpi, cells were fixed and subjected to immunofluorescent by using rabbit anti-HRD1 (red) antibody and mouse anti-E2 antibody (purple). The nuclei were stained with DAPI. Bars = 10 μm. These data are presented as the mean ± SD of data from three independent experiments. *p < 0.05, **p < 0.01, ***p < 0.001.

Previous studies have indicated that endoplasmic reticulum stress induces the expression of METTL14 by inhibiting the ubiquitination of METTL14 mediated by the E3 ubiquitin ligase HRD1 [34]. Since overexpression of NS5B did not increase METTL14 mRNA synthesis, we hypothesized that NS5B might protect METTL14 from degradation, potentially by targeting HRD1 and influencing its regulatory role on METTL14 protein expression. To investigate this, PK-15 cells transfected with pFlag-NS5B were treated with the proteasome inhibitor MG132 (10 μM) and harvested at 3, 6, 9, 12, and 24 hpt for western blotting. The results indicated that overexpression of NS5B significantly protected METTL14 from degradation, leading to increased levels of endogenous METTL14 protein compared to cells transfected with an empty vector (Fig 5A). Further, to confirm whether HRD1 acts as an E3 ubiquitin ligase for METTL14, HEK-293T cells were co-transfected with pEGFP- METTL14 and pHA-HRD1 for 48 h, then harvested and lysed for Co-IP. The results showed that HRD1 co-precipitated with METTL14 (Fig 5B). Additionally, cells, co-transfected with pEGFP-METTL14, pHA-ubiquitin, and pMYC-HRD1, exhibited a significant increase in METTL14 ubiquitination levels upon HRD1 overexpression, suggesting that HRD1 regulated the ubiquitination modification of METTL14 (Fig 5C). To elucidate which polyubiquitin modifications are involved in the degradation of METTL14, HEK-293T cells, co-transfected with pEGFP-METTL14, pHA-HRD1 and pCMV-His-ubiquitin (wild-type or -K6, -K11, -K27, -K29, or -K63), were harvested after 48 hpt and lysed for Co-IP. The results indicated that HRD1 mediated K27-polyubiquitination modification of METTL14 (Fig 5D). Thus, we speculated that NS5B inhibits METTL14 degradation by targeting the E3 ligase activity of HRD1. To determine the inhibitory effect of NS5B on HRD1 E3 ligase activity, HEK-293T cells, co-transfected with the constructs pHA-HRD1 and pFlag-NS5B, were harvested and lysed after 48 hpt for Co-IP assay. The Co-IP results demonstrated an interaction between NS5B and HRD1 (Fig 5E). Moreover, HEK-293T cells co-transfected with pFlag-NS5B, pMYC-HRD1, pHA-ubiquitin, or pEGFP-METTL14 showed a significant reduction in METTL14 ubiquitination in the presence of NS5B (Fig 5F). Confocal fluorescence microscopy was then used to investigate the subcellular localization relationships between NS5B, HRD1, and METTL14 in PK-15 cells. The results suggested that while METTL14 and HRD1 co-located in the nucleus, NS5B and HRD1 were primarily co-located in the cytoplasm (Fig 5G). Cells transfected with pEGFP-METTL14, pFLAG-NS5B, or vector were lysated and subjected to nucleoplasmic separation assay. Interestingly, following the overexpression of NS5B, HRD1 relocated from the nucleus to the cytoplasm. Conversely, the overexpression of METTL14 did not alter the intracellular localization of HRD1 (Fig 5H). These data imply that NS5B might disrupt the co-localization of METTL14 and HRD1 in the nucleus by sequestering HRD1 in the cytoplasm. Further confocal fluorescence microscopy analysis of cells infected with CSFV and transfected with pEGFP-METTL14 revealed co-localization of METTL14 and HRD1 around the nucleus upon infection (Fig 5I). In summary, these findings strongly indicate that HRD1 functions as an E3 ubiquitin ligase for METTL14, but the presence of NS5B appears to hijack HRD1, retaining it in the cytoplasm. This sequestration effectively prevents the ubiquitination of METTL14, resulting in an increased protein expression of METTL14, which is critical for CSFV replication.

### METTL14 targets TLR4 and regulates its degradation

Our results indicate that METTL14 plays a crucial role in augmenting CSFV replication, as evidenced by IHC results which show elevated virus titer in infected tissues characterized by significant pathological damage. This aligns with prior studies indicating that viruses exploit host m^6^A modifications to modulate intracellular metabolic pathways, thereby facilitating their life cycle and establishing a conducive cellular environment for persistent infection.

**Fig 6:**
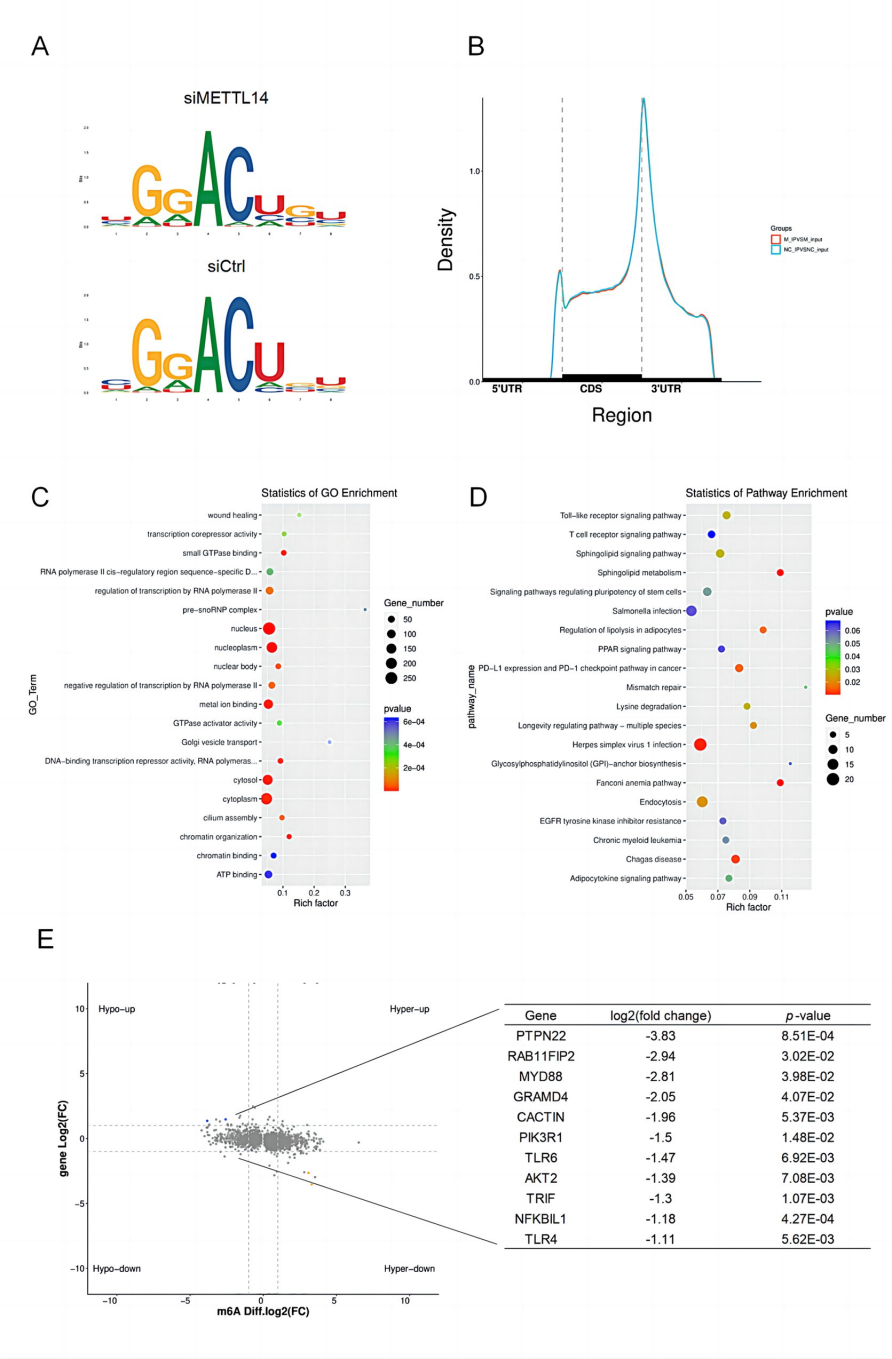
m^6^A modification compares PK-15 cells transfected with siCtrl or siMETTL14 by MeRIP-seq. (A) Sequence logo representing consensus motif of m^6^A sites peaks by MeRIP-seq in PK-15 cells tranfected with siMETTL14 or siCtrl upon CSFV infection (MOI = 1). (B) m^6^A peaks distribution in 5’UTR, stop codon, and 3’UTR region by MeRIP-seq. (C and D) GO and KEGG enrichment analysis in the biological process and signaling pathway category of transcripts bearing m^6^A modification. MeRIP-seq data were from 1 representative of 2 independent experiments. (E) MeRIP-seq results showed that m^6^A peaks of TLR4, TLR6, MYD88, TRIF, and other 7 genes were significantly decreased in METTL14-knockdown PK-15 cells.

Herein, we need to explore the molecular mechanism by which METTL14 promotes CSFV proliferation in the host. To this end, we infected METTL14- knockdown PK-15 cells with CSFV (MOI = 1) and performed MeRIP-seq to analyze alterations in m^6^A modification sites on transcriptome mRNAs at 48 hpi. Interestingly, despite the METTL14 knockdown, the "GGACU" sequence motif, a known site of m^6^A modification on mRNA, remained unaltered, suggesting that METTL14 did not affect the m^6^A modification site in the host during CSFV infection (Fig 6A). Further analysis using MeRIP-seq data to annotate m^6^A peaks on transcriptome mRNA revealed a concentration of these peaks near the 5’UTR, stop codon, and 3’UTR of mRNA (Fig 6B). Gene ontology (GO) enrichment analysis of these differential m^6^A peaks, in conjunction with GO annotations, indicated a primary enrichment of m^6^A peaks in mRNA within the nucleus and nucleoplasm (Fig 6C), which also in accordance with the modification occurring in the nucleus. Subsequent KEGG enrichment analysis pointed to a specific enrichment of m^6^A peaks in pathways related to viral infection and TLR signaling (Fig 6D). Additionally, we identified genes associated with the TLR signaling pathway. As shwon in Fig 6E, a significant reduction in m^6^A peaks was observed on 11 genes, including TLR4, TLR6, MYD88, and TRIF, in cells where METTL14 was knocked down.

**Fig 7:**
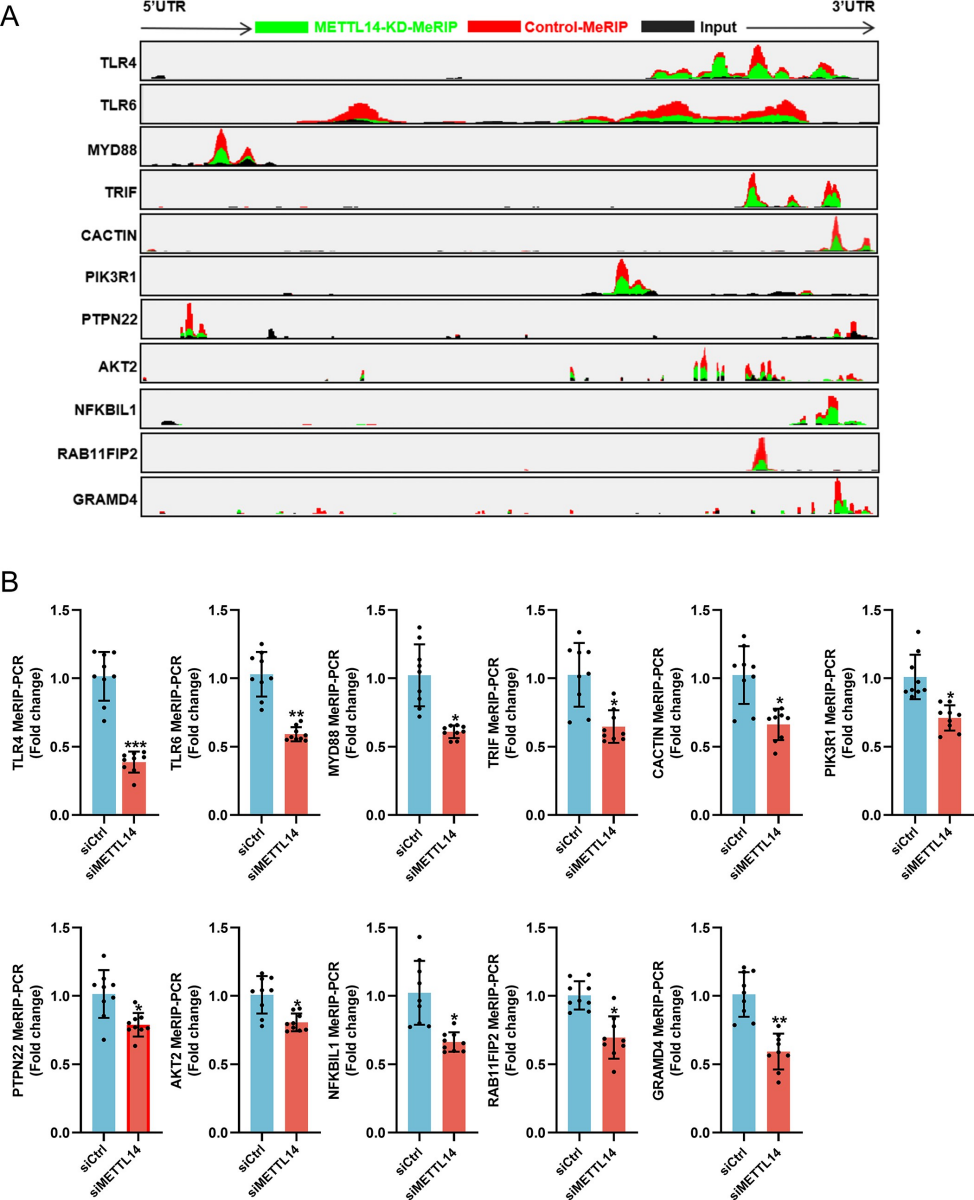
The m^6^A peak of 3’UTR of TLR4 mRNA decreases significantly in METTL14-knockdown PK-15 cells. (A) The m^6^A peak marked in green is decreased in the 3’UTR of TLR4, TLR6, MYD88, TRIF, and other 7 genes from m^6^A-seq data in METTL14-knockdown PK-15 cells infected with CSFV (MOI = 1). Coverage of m^6^A from cells transfected with siCtrl and control reads (Input) were indicated in red and black, respectively. (B) Validation of m^6^A peaks in TLR4, TLR6, MYD88, TRIF, and other 7 genes by MeRIP-RT-qPCR. Fold enrichment was determined by calculating the fold change of IP to input Ct values. IgG precipitated RNA enrichment was used as the control. These data are presented as the mean ± SD of data from three independent experiments. *p < 0.05, **p < 0.01, ***p < 0.001.

Notably, as expected, the m^6^A peaks of 11 genes such as TLR4, TLR6, MYD88, and TRIF are mostly concentrated near the stop codon and 3’UTR [35]. As shown in Fig 7A, the m^6^A peaks of these 11 genes were significantly decreased in METTL14-knockdown cells. This decrease was further validated through MeRIP-RT-qPCR in cells transfected with METTL14-targeting siRNA and infected with CSFV (MOI = 1), harvested at 24 hpi. The results confirmed a substantial reduction in m^6^A peaks on 11 gene mRNAs including TLR4, TLR6, MYD88, and TRIF (Fig 7B). In summary, our findings suggest that METTL14 specifically regulates the m^6^A modification of these 11 genes during CSFV infection, highlighting its pivotal role in viral replication dynamics within the host.

**Fig 8:**
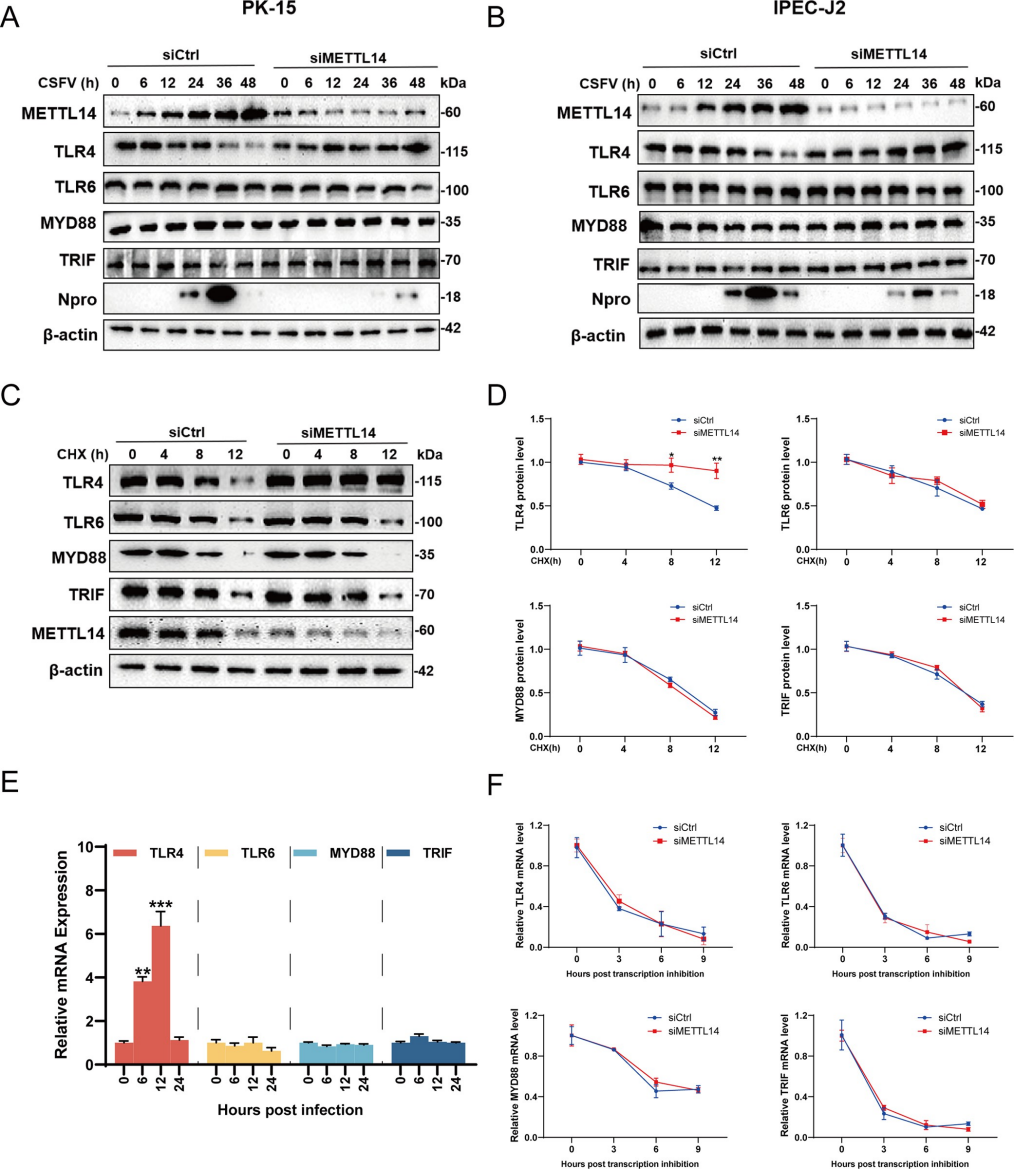
METTL14 reduces the expression of TLR4 during infection. (A and B) PK-15 or IPEC-J2 cells transfected with siMETTL14 or siCtrl were infected with CSFV (MOI = 1) and harvested at indicated time points for western blotting by using rabbit anti-METTL14/TLR4/TLR6/MYD88/TRIF/ Npro antibody and β-actin as a loading control. (C and D) PK-15 cells transfected with siMETTL14 or siCtrl were infected with CSFV (MOI = 1), and then treated with CHX (10 μg/mL). Cells were harvested at indicated times and subjected to western blotting by using rabbit anti-METTL14/TLR4/TLR6/MYD88/TRIF antibody and β-actin as a loading control. These data are representative of three independent experiments. (E) PK-15 cells were infected with CSFV (MOI = 1) and then harvested at indicated times for RT-qPCR. (F) PK-15 cells transfected with siMETTL14 or siCtrl were infected with CSFV (MOI = 1) and then treated with actinomycin D (5 μg/mL). Cells were harvested at indicated times and subjected to RT-qPCR. These data are presented as the mean ± SD of data from three independent experiments. *p < 0.05, **p < 0.01, ***p < 0.001.

Hence, have the protein levels of METTL14-methylated TLR4, TLR6, MYD88, and TRIF genes been changed? PK-15 and IPEC-J2 cells transfected with siRNA targeting METTL14 or siCtrl for 24 h were infected with CSFV (MOI = 1), then harvested at 6, 12, 24, 36, and 48 hpi and lysed for western blotting. The findings revealed a notable increase in TLR4 protein expression in METTL14-knockdown infected PK-15 and IPEC-J2 cells, while TLR6, MYD88, and TRIF protein levels remained largely unchanged (Fig 8A and 8B). Further investigation into role of METTL14 in regulating the protein levels of TLR4, TLR6, MYD88, and TRIF was conducted. Cells transfected with siRNA targeting METTL14 or siCtrl for 24 h were infected with CSFV (MOI = 1) and treated with CHX (10 μg/mL). Subsequent harvesting at 4, 8, and 12 hpt for western blotting indicated that METTL14 knockdown hindered the degradation of TLR4, while leaving the protein expressions of TLR6, MYD88, and TRIF unaffected (Fig 8C and 8D). Then, to further determine whether knockdown of METTL14 affects the mRNA generation or degradation rate of TLR4, TLR6, MYD88, and TRIF. The infected cells were collected and lysed at 6, 12, and 24 hpi for RT-qPCR. The result showed an increase in TLR4 mRNA synthesis after 6 and 12 hpi, but no significant change after 24 hpi. Conversely, there were no changes in mRNAs generation levels of TLR6, MYD88, and TRIF after CSFV infection (Fig 8E). Additionally, PK-15 cells transfected with METTL14- targeting siRNA for 24 h and infected with CSFV (MOI = 1) were treated with actinomycin D (5 μg/mL) at 24 hpi. Cells were then collected at 3, 6, and 9 hpt for RT-qPCR. The results showed that knockdown of METTL14 did not affect the mRNA half-lifes of TLR4, TLR6, MYD88, and TRIF in infected cells (Fig 8F). In summary, the compiled data suggest that while METTL14 does not significantly impact the mRNA half-life of TLR4, it plays a crucial role in regulating its degradation.

### YTHDF2 is responsible for TLR4 degradation

**Fig 9:**
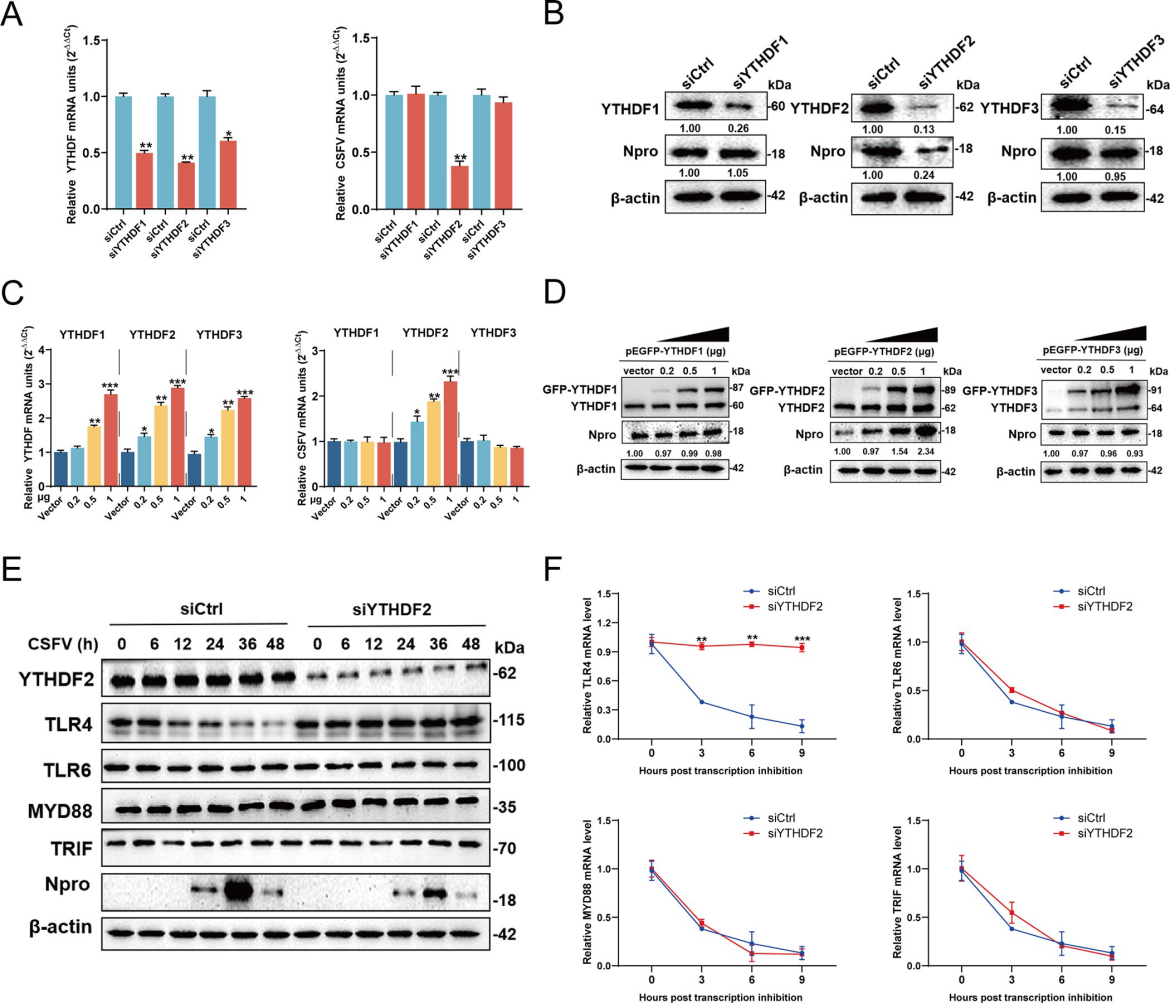
YTHDF2 induces the decay of TLR4 mRNA during infection. (A-D) PK-15, IPEC-J2, and ST cells were transfected with siYTHDF1-3 or siCtrl or pEGFP-YTHDF1-3 (0.2, 0.5 and 1 μg) and infected with CSFV (MOI = 1). At 24 hpi, the total RNA in treated cells were extracted and subjected to RT-qPCR for detecting the mRNA levels of target genes (A and C). Alternatively, cells were harvested and subjected to western blotting using the indicated antibodies against YTHDF1, YTHDF2, YTHDF3, Npro, and β-actin for detecting the expression levels of target proteins (B and D). (E) PK-15 cells transfected with siYTHDF2 or siCtrl were infected with CSFV (MOI = 1). Cells were harvested at indicated times and subjected to western blotting by using rabbit anti-YTHDF2/TLR4/TLR6/MYD88/TRIF/Npro antibody, along with β- actin as a loading control. (F) PK-15 cells transfected with siYTHDF2 or siCtrl were infected with CSFV (MOI = 1) and then treated with actinomycin D (5 μg/mL). Cells were harvested at indicated times and subjected to RT-qPCR. These data are presented as the mean ± SD of data from three independent experiments. *p < 0.05, **p < 0.01, ***p < 0.001.

Previous studies have established that methylated mRNAs are translocated to the cytoplasm where they associate with reader proteins (YTHDF1, YTHDF2, and YTHDF3), influencing RNA processes such as alternative splicing, translation, stability, or cellular localization [36–39]. Our studies demonstrated that METTL14 specifically modified the mRNAs of TLR4, TLR6, MYD88, and TRIF. However, METTL14 knockdown did not impact the half-lifes of these mRNAs. Consequently, we speculated whether YTHDF proteins played a role in maintaining the steady states of these mRNAs. To test this hypothesis, PK-15 cells were transfected with siRNAs targeting YTHDF1-3 or siCtrl and then infected with CSFV (MOI = 1). At 24 hpi, cells were harvested for RT-qPCR and western blotting. The results indicated that YTHDF2 knockdown significantly reduced viral mRNA and Npro protein levels, while YTHDF1 and YTHDF3 knockdowns had no impacts (Fig 9A and 9B). Further experiments involved transfecting PK-15 cells with varying concentrations of pEGFP- YTHDF1-3 constructs for 24 h, followed by CSFV infection (MOI = 1). Analysis post 24 hpi showed that overexpressing YTHDF2 notably increased viral mRNA synthesis and Npro protein expression, whereas overexpression of YTHDF1 or YTHDF3 had no effects (Fig 9C and 9D). Intriguingly, YTHDF2 knockdown also elevated TLR4 protein expression in infected cells, with no significant effects on TLR6, MYD88, and TRIF protein levels (Fig 9E). Given that YTHDF2 is known to recognize m^6^A methylated RNA in the cytoplasm and influence its half-life [40], we investigated if YTHDF2 mediated RNA degradation. Cells with YTHDF2 knockdown, infected with CSFV (MOI = 1) and treated with actinomycin D to halt RNA synthesis, were analyzed via RT-qPCR at different time points. The findings revealed increased stability of TLR4 RNA in YTHDF2-knockdown cells, whereas the decay rates of TLR6, MYD88, and TRIF RNAs remained unchanged (Fig 9F). The findings are critical, illustrating that METTL14-mediated methylation of TLR4 mRNA is specifically targeted by YTHDF2 in the cytoplasm, leading to its degradation.

**Fig 10:**
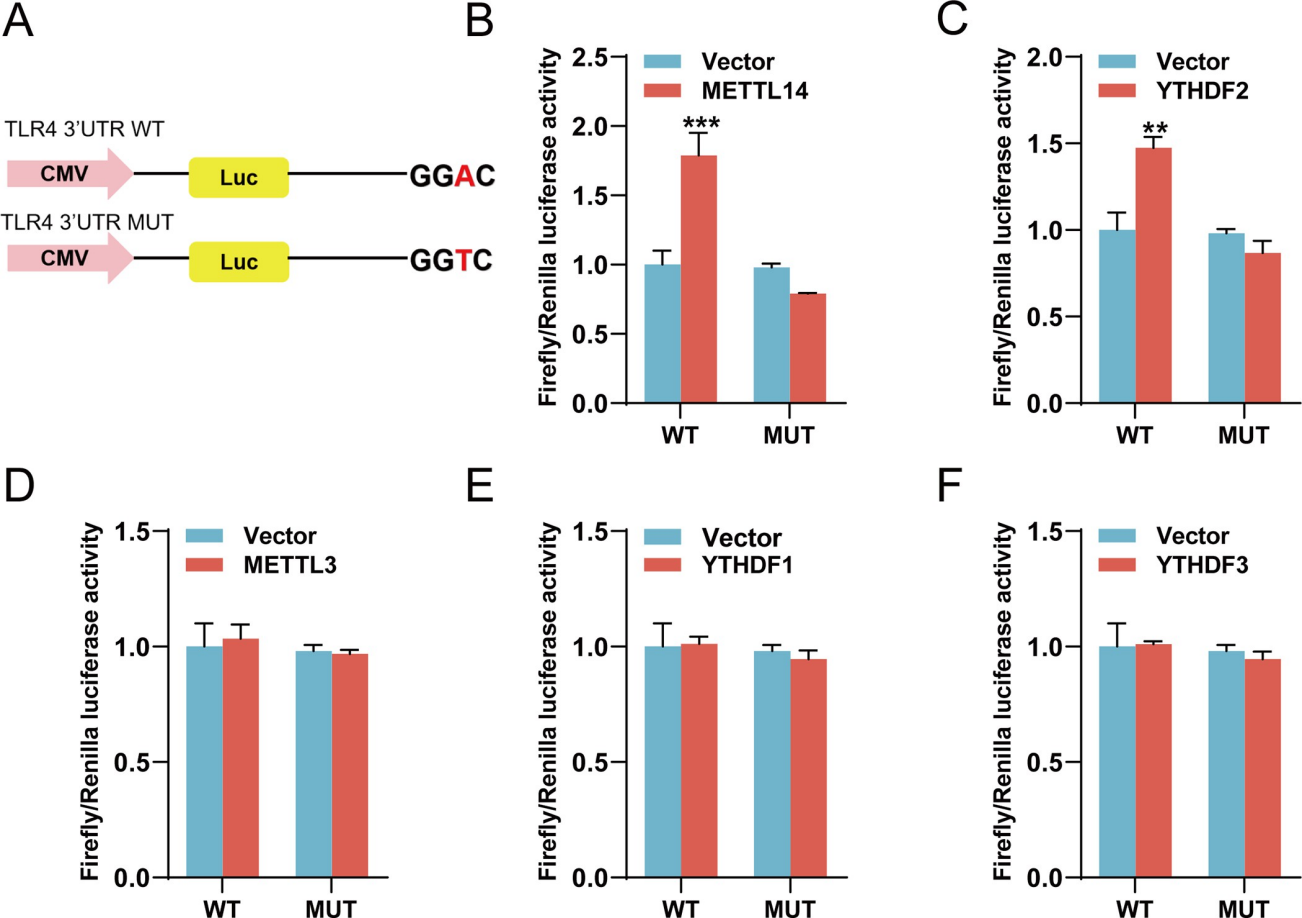
METTL14 and YTHDF2 recognize the m^6^A site on 3’UTR of TLR4 mRNA. (A) Schematic representation of mutated (“GGACT” to “GGCCT”, red dots) 3’UTR of pGL3-Basic vector to investigate the m^6^A roles on TLR4 expression. (B-F) PK-15 cells were co-transfected with pGL3-TLR4-WT or the corresponding mutants, pEGFP-YTHDF1, -YTHDF2, -YTHDF3, -METTL3 or -METTL14, and PRL-TK, respectively. The total mRNA in transfected cells were extracted and subjected to Luciferase reporter assay. These data are presented as the mean ± SD of data from three independent experiments. **p < 0.01, ***p < 0.001.

Confirming the accuracy of the m^6^A modification site (258053820- 258054641nt, *chromosome* 1) on the 3’UTR of TLR4 mRNA is of paramount importance. To this end, we engineered the pGL3-TLR4 constructs, which incorporated either the wild-type sequence of the TLR4 3’UTR (designated as TLR4-WT) or a mutated version (TLR4-MUT), where adenine was substituted with thymine (Fig 10A). The luciferase reporter assay revealed that METTL14 and YTHDF2 overexpression markedly enhanced the luciferase activity of TLR4-WT, but exerted no influence on TLR4-MUT (Fig 10B and 10C). Conversely, overexpression of METTL3, YTHDF1, and YTHDF3 did not impact either TLR4-WT or TLR4-MUT (Fig 10D, 10E and 10F). Collectively, these findings suggest that the regulation of TLR4 mRNA stability by METTL14 and YTHDF2 is contingent upon the m^6^A modification site within the 3’UTR, which in turn, leads to a reduction in METTL14 protein expression.

### METTL14 inhibits TLR4-regulated NF-κB signaling during infection

**Fig 11:**
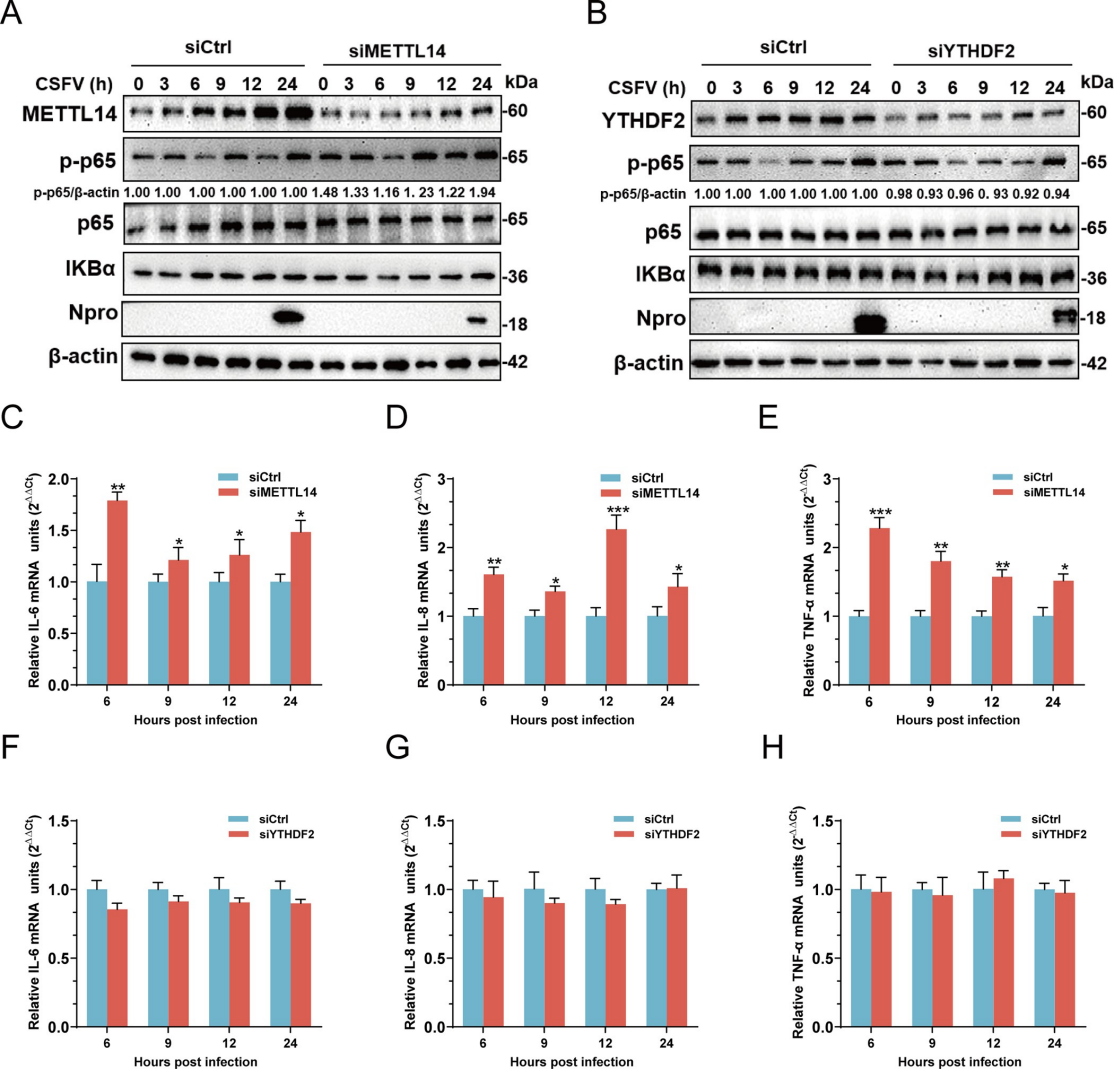
METTL14 inhibits TLR4 regulated NF-κB pathway during infection. (A and B) PK-15 cells transfected with siMETTL14 (A), siYTHDF2 (B), or siCtrl were infected with CSFV (MOI = 1). Cells were harvested at indicated times and subjected to western blotting by using rabbit anti-METTL14/YTHDF2 /p-p65/p65/IKBα/Npro antibody and β-actin as a loading control. (C-H) PK-15 cells transfected with siYTHDF2, siMETTL14 or siCtrl were infected with CSFV (MOI = 1). The total mRNA in transfected cells at indicated times were extracted and subjected to RT-qPCR for detecting the mRNA levels of inflammatory cytokines. These data are presented as the mean ± SD of data from three independent experiments. *p < 0.05, **p < 0.01, ***p < 0.001.

Previous research has established that m^6^A modification modifies antiviral transcripts and influences the host innate immunity [41]. Furthermore, it has been observed that the activation of NF-κB signaling in PK-15 cells impedes CSFV replication, underscoring the virus sensitivity to NF-κB-induced inflammatory cytokines. Meanwhile, TLR4 is known to trigger NF-κB or IFN-I/III signaling through MYD88 and TRIF [42,43]. In our study, we sought to assess whether the knockdown of METTL14 or YTHDF2 affected NF-κB or IFN-I/III signaling during CSFV infection. PK-15 cells, transfected with siRNAs targeting METTL14 or YTHDF2 for 24 h, were infected with CSFV (MOI = 1). At various time points post-infection, the cells were collected and processed for western blotting. The results indicated that METTL14 knockdown significantly augmented the phosphorylation of p65 protein, a key marker of NF-κB signaling, without altering the protein levels of p65 and IKBα, which ultimately reduced Npro protein expression (Fig 11A). Conversely, while YTHDF2 knockdown also diminished Npro protein expression, it did not influence the protein expressions of p-p65, p65, and IKBα (Fig 11B). Consistent with the western blotting findings, METTL14 knockdown notably increased the mRNA levels of IL-6, IL-8, and TNF-α in infected cells (Fig 11C, 11D and 11E). Conversely, YTHDF2 knockdown had no impact on the mRNA levels of these cytokines in infected cells (Fig 11F, 11G and 11H).

To elucidate the regulatory role of TLR4 on the NF-κB signaling pathway during CSFV infection, cells transfected with siRNA targeting TLR4 for 24 h were infected with CSFV (MOI = 1). Cell lysates were collected at 3, 6, 9, 12, and 24 hpi and subjected to RT-qPCR and western blotting assays. The results demonstrated that TLR4 knockdown significantly impeded the protein expressions of p-p65, p65, and IKBα (S3A Fig) and mRNA levels of IL-6, IL-8, and TNF-α in infected cells during CSFV infection (S3B–S3D Fig). These results suggested that TLR4 played a critical role in specifically inducing NF-κB to counteract CSFV replication. These results contribute to a deeper understanding of the mechanism by which METTL14, as opposed to YTHDF2, critically regulates the NF-κB signaling pathway, thereby facilitating CSFV infection. Furthermore, we observed that METTL14 knockdown did not affect the protein expressions of p-TBK1, TBK1, p-IRF3, IRF3, p-STAT1, STAT1, Mx1, and ISG15 in CSFV-infected cells (S4A Fig). RT-qPCR analysis revealed that the mRNA generations of STAT1, TBK1, IRF3, IFNα, IFNβ, Mx1, and ISG15 remained unchanged in METTL14- knockdown cells upon CSFV infection (S4B Fig). Overall, these findings indicate that METTL14 suppresses the NF-κB signaling pathway at its origin, without affecting the IFN signaling pathway, thereby facilitating the replication of CSFV.

## Discussion

While m^6^A methylation sites on viral genome RNA were identified as early as the 1970s, significant insights into the role of m^6^A methylation in modulating host metabolism and influencing viral infection have only begun to emerge in recent years [44–46]. Studies have shown that viral infections lead to changes in the m^6^A modifications of the host cell transcriptome, which may either enhance viral replication or strengthen the host defense against the infection [2,21].

METTL3 primarily catalyzes the transfer of methyl groups from SAM (S-adenosyl methionine, SAM) to adenine bases within the methyltransferase complex, resulting in the production of SAH (S-adenosyl homocysteine, SAH). METTL14, on the other hand, is predominantly involved in stabilizing the structure of these complexes and selecting specific RNA sequences as catalytic substrates [17]. Our studies have for the first time established that infection with a virulent strain of CSFV upregulates both METTL14 protein expression and m^6^A methylation levels *in vivo* and *in vitro*. Interestingly, infections by other viruses like BVDV, PRV, and JEV did not induce similar changes in METTL14 expression, indicating that it was a specific alteration triggered by CSFV infection. This upregulation of m^6^A methylation levels may be directly linked to the increased expression of METTL14.

Additionally, studies have shown that the detection rates of CSFV in peripheral lymphatic organs (such as the tonsil, lymph node, and spleen) are significantly higher than in other organs, and the virus has also been isolated from the kidney. Consistent with these findings, our study demonstrated high virus titers in the lymph node, spleen, and kidney. This correlates with increased METTL14 expression and elevated m^6^A modification levels in these specific organs. Such an association further underscores the connection between the heightened presence of CSFV and the increase in both METTL14 expression and m^6^A modifications, highlighting the specific influence of virus on these molecular pathways.

Previous study has reported that METTL3 impedes the degradation of IRAKM, a key negative regulator of TLR4, consequently leading to the suppression of TLR4 and its associated downstream signaling pathways [47]. METTL14 collaborates with METTL3 in regulating the replication of various viruses, including HCV [48], HDV [49], IAV [50], HSV-1 [51] and HIV [52], suggesting a supporting role for METTL14 in these viral infections. However, in the case of viruses like PCV2 [53], PRV [54], VSV [55], and EBV [24], METTL14 is shown to play a crucial role in promoting their replication. Our findings are aligned with this observation, demonstrating that the knockdown of METTL14 notably impedes CSFV replication, while its overexpression leads to an increase in viral load. In contrast, the manipulation of METTL3 levels, either through knockdown or overexpression, did not impact CSFV replication. Specifically, SAH, an inhibitor of the METTL3-14 heterodimer complex, significantly reduces the mRNA and protein levels of METTL14 in treated cells, without affecting METTL3 [56]. This was further corroborated by drug inhibition studies, which indicated that

### METTL14 is a necessary component for CSFV replication

EV71 infection significantly upregulates the protein expression levels of METTL3/METTL14. The interaction between EV71 RdRp 3D and METTL3 enhances the SUMOylation and ubiquitination of RdRp 3D, thereby increasing EV71 replication [57]. HRD1 could mediates the ubiquitination and degradation of METTL14 by ERAD [34]. Our results contributed to this understanding by revealing the interaction between the E3 ligase HRD1 and its substrate, METTL14. This interaction leaded to the ubiquitination of METTL14. NS5B, a viral protein, competitively binded to HRD1 along with METTL14, hindering the ubiquitination and subsequent degradation of METTL14 by HRD1. This interference results in elevated protein levels of METTL14, further emphasizing the intricate molecular interplay between viral proteins and host cellular mechanisms in the context of CSFV replication.

In the realm of post-transcriptional gene regulation involving m^6^A, various m^6^A-binding proteins are known to recognize m^6^A-modified transcripts, influencing their splicing, export, translation, and degradation in RNA metabolism [36–39]. Specifically, YTHDF2 is adept at selectively identifying m^6^A sites and recruiting the CCR4-NOT deadenylase complex to initiate mRNA degradation in processing bodies, thereby impacting mRNA stability [40]. Mechanistically, METTL14 introduced m^6^A modifications to TLR4 mRNA, promoting degradation of the corresponding protein, without affecting mRNA decay. Intriguingly, YTHDF2 indirectly boosted CSFV replication. Our data implied that YTHDF2 mediated the degradation of TLR4 mRNA, resulting in diminished TLR4 protein levels.

TLRs activation by ligands initiates two signaling cascades: the MYD88- dependent pathway, leading predominantly to the production of pro-inflammatory cytokines and chemokines, and the TRIF-dependent pathway, which generates IFN and pro-inflammatory cytokines. Viral infections activate TLR4, thereby triggering subsequent cascade reactions that activate NF-κB and IFN signaling pathways [58–60]. Our studies revealed that CSFV infection dampened NF-κB signaling and inflammatory cytokines production by diminishing TLR4 protein expression through METTL14. Importantly, METTL14 knockdown left the IFN pathway unaffected. Additionally, while YTHDF2 promoted CSFV replication, it did not influence NF-κB signaling. These findings highlight critical role of METTL14 in modulating CSFV life cycle via host methylation.

**Fig 12:**
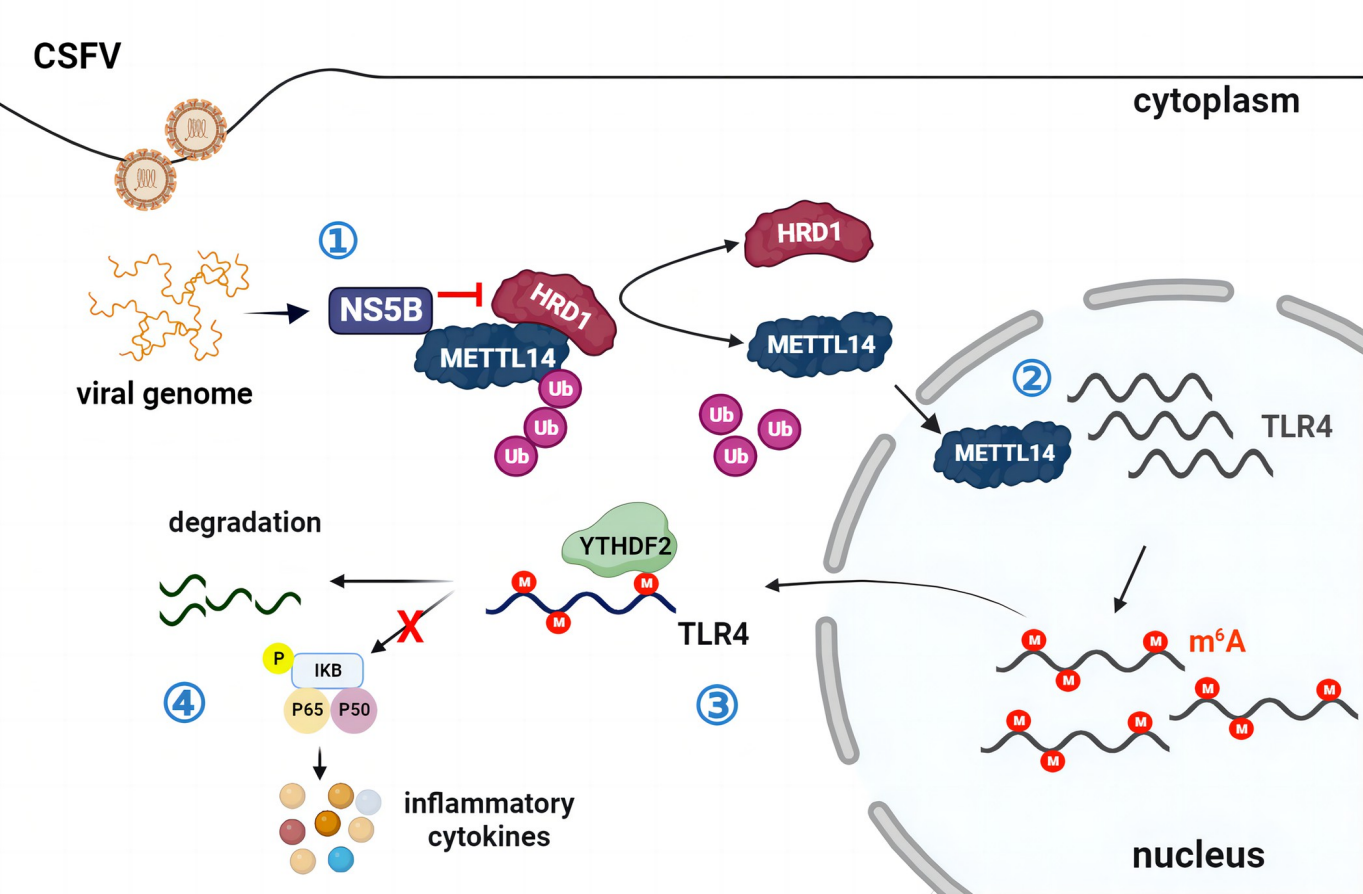
The proposed schematic model of METTL14 in TLR4-mediated innate immunity and promoting CSFV replication. (1) CSFV nonstructural protein NS5B hijacks HRD1 to inhibit its ubiquitination modification of METTL14. (2) During CSFV infection, upregulated METTL14 mediates the m^6^A modification in TLR4 mRNA and (3) recruits YTHDF2 that recognizes and affects the modified TLR4 mRNA decay, ultimately leading to a reduction in TLR4 protein expression, (4) followed by the inhibition of NF-κB signaling pathway.

Summarizing, as shown in Fig 12, the CSFV NS5B protein was instrumental in commandeering HRD1 and inhibiting its ubiquitination modification of METTL14. CSFV then mediates the m^6^A modification in TLR4 mRNA via METTL14, and YTHDF2 recognized and accelerated the decay of the modified TLR4 mRNA, leading to a decrease in TLR4 protein expression and consequent inhibition of the NF-κB pathway, thereby fostering CSFV replication. Our study highlighted the critical role of METTL14 in the methylation process that regulated the CSFV life cycle. The post- transcriptional m^6^A regulation on specific mRNA emerges as a crucial factor in viral infection dynamics. These insights position METTL14 as a potential therapeutic target in combating CSFV infection. Unraveling these mechanisms will provide a deeper comprehension of CSFV pathogenicity.

## Materials and methods

### Ethics statement

All animal experiments in this study received approval from the Animal Ethics Committee of Nanjing Agricultural University and adhered to the “Guidelines on Ethical Treatment of Experimental Animals” (2006), No. 398, as stipulated by the Ministry of Science and Technology, China. The animals were accommodated at the Animal Experiment Center of Nanjing Agricultural University in compliance with these guidelines. We ensured diligent efforts to minimize distress, pain, or discomfort to the animals throughout the experimental procedures.

### Virus, cells and plasmids

Human Embryonic Kidney 293 (HEK-293T), Porcine Kidney (PK-15), Intestinal Porcine Epithelial Cell-J2 (IPEC-J2), and Swine Testicle (ST) cells were cultured in Dulbecco’s Modified Eagle’s Medium (DMEM, GIBCO, Invitrogen, Carlsbad, CA, USA) supplemented with 10% fetal bovine serum (FBS, GIBCO, Invitrogen), 0.2% NaHCO_3_, 100 μg/mL streptomycin, and 100 IU/mL penicillin (GIBCO, Invitrogen). Classic Swine Fever virus (CSFV) Shimen strain (GenBank accession number: AF092448) and vaccine virus (HCLV) strain (GenBank accession number: AF531433) were kept in the lab. Genes YTHDF1, YTHDF2, YTHDF3, METTL3, and METTL14 were cloned into pEGFP-C1 for overexpression studies. Plasmids pFlag-E2, -Core, -NS3, -NS4B, -NS5A, and -NS5B were created by cloning corresponding CSFV genes into the p3×Flag-CMV-7.1 vector. The HRD1 gene was cloned into pCMV-MYC and pcDNA3.0 to yield pMYC-HRD1 and pHA-HRD1 constructs. Ubiquitin gene was cloned into pcDNA3.0 and pCMV-His to produce pHA-ubiquitin and pCMV-His-ubiquitin (WT and -K6, -K11, -K27, -K29, or -K63). The 3’UTR of TLR4 and its mutants were cloned into pGL3-basic to generate pGL3-TLR4-WT or pGL3-TLR4-MUT constructs. All plasmids were verified through DNA sequencing.

### Plasmids and siRNA transfections

PK-15, IPEC-J2, and ST cells, at 70% confluence, were transfected with specified plasmids using jetPRIME transfection reagent (Polyplus, France) as per the manufacturer’s protocol. For RNA interference, these cells were transfected with siRNA using INTERFERin transfection reagent (Polyplus, France), following the manufacturer’s guidelines. The siRNA duplexes and control siRNA, synthesized by Gene Pharma (GenePharma, China), are listed in **S1 Table**. Post 24 hours of transfection (hpt), cells were infected with CSFV and analyzed for intended gene expression (details in **S2 Table**) and virus replication via RT-qPCR or western blotting at 24 hpi.

### Quantitative RT-PCR (RT-qPCR)

RT-qPCR was performed as described previously [61]. The target mRNA was quantified using the primers indicated in **S2 Table**. Data was presented as 2^-ΔΔCT^ from quadruplicate samples [62].

### Virus titers in the tissues

A mass of 1 gram was meticulously weighed from tissues extracted from lymph node, spleen, kidney, lung, tonsil, heart, and small intestine. These specimens were subsequently subjected to pulverization into fine fragments under the application of liquid nitrogen, followed by resuspension in a volume of 500μl PBS. The resuspended specimens were then processed for RNA extraction employing the TRIzol reagent and sent to RT-qPCR as follow: serial dilutions of the CSFV nucleic acid were prepared, ranging from 10^1^ to 10^6^, to facilitate the establishment of a standard curve. The ultimate load of the CSFV per gram of tissue was ascertained by referencing this standard curve against the control standards of the assay.

### Cell viability assay (CCK-8)

PK-15 cells plated in a 96-well plate at a density of 10^4^ cells per well were exposed to varying concentrations of pharmacological agents for a duration of 24 h. The cytotoxic impact of these agents on the cells was quantitatively assessed utilizing the CCK-8 assay kit (abs50003, Absin Bioscience, Inc., China). Following a 4 h incubation period at 37°C, the fluorescence emitted by the agent was measured using a fluorescence microplate reader. The results indicated an absence of cytotoxic effects in cells treated with the specified concentrations of drug duplexes.

### Cytoplasmic and nuclear protein extraction

PK-15 cells seeded on 6-well plates, a density of 70%, were transfected with pFLAG-NS5B, pEGFP-METTL14, or a vector using jetPRIME transfection reagent (Polyplus, France) as per the manufacturer’s protocol. After 24 h, total proteins from the cytoplasm and nucleus in treated cells were separately extracted using a Nuclearand Cytoplasmic Protein Extraction Kit (BB36021, BestBio, China) according to the manufacturer’s protocol. These proteins were then subjected to western blotting for further identification.

### Virus titration

PK-15 cells, after undergoing transfection, were infected with the CSFV Shimen strain for a duration of 1 hour. Subsequently, at 24 or 48 hpi, cells were subjected to lysis through three cycles of freezing and thawing. The yields of the virus were quantified using the assay previously described [62]. The data are presented as the tissue culture infectious dose 50 (TCID_50_) calculated from quadruplicate samples.

### Co-immunoprecipitation (Co-IP) and western blotting

Initially, to study interactions between CSFV protein and METTL14, HEK-293T cells were co-transfected with pEGFP-METTL14 and plasmids (pFlag-Core, -NS3, -NS4B, -NS5A, -NS5B, or -E2) at 37°C for 48 h. Cells were lysed in NP-40 buffer (50 mM Tris-HCl, 150 mM NaCl, 1% NP-40, 1 mM EDTA, 1 mM PMSF, 1 mM NaF, 1 mM Na_3_VO_4_, pH 7.4) for 30 min at 4°C. After centrifugation (1,000×g for 10 min at 4°C), 20% of the supernatant was reserved for analysis. The remaining lysate was incubated with control IgG and Protein A/G PLUS-Agarose slurry (sc-2003, Santa Cruz, USA) for 4 h at 4°C. Post-removal of agarose beads by centrifugation, lysates were incubated with anti-Flag antibody (F1804, Sigma, USA) and further with Protein A/G PLUS- Agarose for 2 h. The beads were washed with NP-40 buffer and prepared for SDS-PAGE and western blotting. Immunoprecipitations and lysates were analyzed using rabbit anti-METTL14 (26158-1-AP, Proteintech, China), mouse anti-Flag (F1804, Sigma, USA), and rabbit anti-β-actin antibody (AC038, ABclonal, China). To explore HRD1-METTL14 and HRD1- NS5B interactions, HEK-293T cells were co-transfected with pHA-HRD1 and pEGFP-METTL14 or pHA-HRD1 and pFlag-NS5B for the same experiment operation as described above after 48 hpt. Co-IP and whole cell lysates were harvested and subjected to western blotting by using rabbit anti-METTL14 antibody, mouse anti-Flag antibody, rabbit anti-HA antibody and rabbit anti-β-actin antibody.

### Co-IP and Ubiquitination

HEK-293T cells transfected with pHA-Ubiquitination, pEGFP-METTL14, or pMYC-HRD1 were lysed in NP-40 buffer after 48 hpt for Co-IP assay. Cell lysates were processed for western blotting using rabbit anti-METTL14/MYC antibody (Proteintech, China), rabbit anti-HA antibody (Sigma, USA), and rabbit anti-β-actin antibody (ABclonal, China). Additionally, to examine effect of NS5B on HRD1 E3 ligase activity, HEK-293T cells transfected with pHA-Ubiquitination, pEGFP-METTL14, pFlag-NS5B, or pMYC-HRD1 were lysed in NP-40 buffer after 48 hpt for Co-IP assay. Cell lysates were processed for western blotting using rabbit anti-METTL14/MYC antibody (Proteintech, China), rabbit anti-HA antibody (Sigma, USA), and rabbit anti-β-actin antibody (ABclonal, China). For studying polyubiquitination of METTL14, cells transfected with pHis-Ub (WT and -K6, -K11, -K27, -K29, or -K63), pEGFP- METTL14, and pHA-HRD1. At 48 hpt, cells were lysed in NP-40 lysis buffer for the same experiment operation as above. Cell lysates were processed for western blotting using rabbit anti- METTL14/His antibody (Proteintech, China), rabbit anti-HA antibody, and rabbit anti-β-actin antibody.

### Confocal fluorescence microscopy

To observe the co-localization of CSFV proteins with METTL14, PK-15 cells cultured on dishes were co-transfected with pEGFP-METTL14 and pFlag-Core, NS3, NS4B, NS5A, NS5B, or E2, and then incubated at 37°C for 24 h. Post-incubation, cells were fixed with 4% paraformaldehyde (PFA) in PBS and permeabilized with 0.1% Triton X-100 before confocal microscopy analysis using a mouse anti-Flag antibody (Sigma, USA). For confirming the co-localization of HRD1 with METTL14 or NS5B, similarly prepared PK-15 cells were transfected with pEGFP-C1, pEGFP-METTL14, or pFlag-NS5B and subjected to confocal microscopy using rabbit anti-HRD1 (A2605, ABclonal, China) and mouse anti-Flag (Sigma, USA) antibodies. Additionally, to examine HRD1 and METTL14 co-localization during CSFV infection, PK-15 cells transfected with pEGFP-METTL14 and infected with CSFV or not (MOI = 1) at 37°C for 24 hpi were fixed and analyzed by confocal microscopy using rabbit anti-HRD1 and mouse anti-CSFV E2 antibodies (WH303). Co-localization coefficients were calculated using Image J 7.0 software.

### Animal experiment

Ten six-week-old specific-pathogen-free Large White pigs were randomly divided into two groups of five and housed in separate rooms at the Animal Experiment Center of Nanjing Agricultural University. Prior to the experiments, serological tests confirmed the pigs were free from key pathogens such as CSFV, PRRSV, PCV2, FMDV, PRV, using ELISA kits from IDEXX Laboratories, Inc. and JBT Agency, complemented by PCR/RT-PCR assays for virus detection. The infected group was challenged oro-nasally with the CSFV Shimen strain (10^5^ TCID_50_), while the negative control (NC) group received saline. Daily assessments included rectal temperature, clinical scoring (1 point for no fever, 2 for mild clinical signs with pyrexia, 3 for severe clinical signs, 4 for mortality), and pathological examinations, following established protocols [63]. On 9 dpc, pigs exhibiting typical symptoms like high fever, diarrhea, and skin hemorrhages were euthanized. Tissues including lymph nodes, spleen, kidney, lung, tonsil, heart, and intestine were collected for RT-qPCR, western blotting, and Immunohistochemistry (IHC) assays.

### Immunohistochemistry (IHC)

Paraffin-embedded tissue sections were placed on glass slides and dried overnight at 37°C. Following deparaffinization, antigen retrieval was conducted in 0.01 M citric acid buffer. Endogenous peroxidase activity was quenched with 3% H_2_O_2_ in methanol for 10 min. Tissues were then blocked with 3% BSA for 30 min at room temperature. Sections were incubated with a 1:1000 dilution of anti-METTL3/METTL14 antibody (Proteintech, China) overnight at 4°C. An appropriate amount of HRP-conjugated secondary antibody was applied, followed by a 30-min incubation at room temperature. Tissues were stained with DAB solution for 2 min and counterstained with hematoxylin for 2 min. After washing, slides were dehydrated with ethanol, cleared with xylene, and examined under a microscope.

### Quantification of the m^6^A level

Total RNA was extracted using TRIzol and treated with DNase (Sigma, USA). RNA quality and concentration were assessed using NanoDrop. The m^6^A levels in mRNA were measured using the EpiQuik m^6^A RNA Methylation Quantification Kit (EpiGentek, USA) according to the manufacturer’s instructions. Absorbance was read at 450 nm and calculations were based on a standard curve.

### MeRIP-seq assay

PK-15 cells on 15 cm plates, transfected with siRNA targeting METTL14, were infected with CSFV (MOI = 1) at 48 hpt. At 48 hpi, RNA was extracted using TRIzol (TAKARA, Japan), treated with TURBO DNase I (Thermo Fisher, USA), and mRNA was isolated using the Dynabeads mRNA Purification Kit (Thermo Fisher, USA). The mRNA was concentrated via ethanol precipitation. RNA samples were sent to LC-Bio (Hangzhou, China) for sequencing. RNA-seq libraries, prepared from both eluates and 10% input mRNA using the TruSeq mRNA library prep kit (Illumina), underwent quality control (MultiQC) and were sequenced on a HiSeq 4000 instrument.

### MeRIP-RT-qPCR assay

PK-15 cells seeded in 6-well plates were transfected with siRNA targeting METTL14 and infected with CSFV (MOI = 1). At 48 hpi, the total RNA was extracted by using TRIzol reagent (TAKARA, Japan). 20–50 μg fragmented RNA was precleared with 40 μL protein A/G agarose beads (Santa Cruz, USA) supplemented with 40U RNase inhibitor overnight at 4°C. The mixture and 2 μg m^6^A antibody (A19841, ABclonal, China) were incubated overnight at 4°C. A negative control was included with normal IgG (Cell Signaling Technology, USA). Protein A/G agarose beads were added to capture the immunoprecipitated complexes. RNA was eluted from the beads by incubation in 500 μL of elution buffer (5 mM Tris-HCl, pH 7.4; 1 mM EDTA, pH 8.0 and 0.05% sodium dodecyl sulfate) with 20 mg of proteinase K for 1 h at 60°C. Following ethanol precipitation, the input and m^6^A-enriched RNA were reversely transcribed with random primer, and the gene enrichment was determined by RT-qPCR. The primers of m^6^A-enriched gene mRNAs used in this study were shown in **S2 Table**.

### RNA decay assay

PK-15 cells seeded in 24-well plates were transfected with siRNA targeting METTL14 or YTHDF2 and infected with CSFV (MOI = 1), following by the treatment with actinomycin D at a final concentration of 5 μg/mL for the indicated time points. Cells were harvested and the total RNA was extracted to detect mRNA levels of TLR4, TLR6, MYD88, TRIF, and other 7 genes by RT-qPCR. The data were normalized to the time point t = 0.

### Luciferase reporter assay

PK-15 cells seeded on 24-well plates were co-transfected with 300ng pGL3-basic-TLR4 or the corresponding mutants, 300 ng pEGFP-YTHDF1, -YTHDF2, -YTHDF3, -METTL3, or -METTL14, and 50 ng PRL-TK, respectively. Renilla and Firefly luciferase activities were measured at 24 hpt using by the Dual-Luciferase Reporter Assay System (DD1205-01, Vazyme, China). Two- tailed t tests were used to measure statistical significance of differences in reporter expression.

### Statistical analysis

All data were presented as means ± the standard deviations (SD). A student t-test was used to compare the data from the pairs of treated and untreated groups. Statistical significance was indicated by asterisks (* *P*< 0.05; ** *P* < 0.01; *** *P* < 0.001) in the figures. All statistical analyses and calculations were performed using Prism 8 (GraphPad Software, Inc, LaJolla, CA).

## Supporting information

S1 Fig METTL3 level is not changed *in vivo*. (A) Lymph node, spleen, kidney, lung, tonsil, heart, and intestine from CSFV-infected or PBS-treated pigs were extracted RNA or lysed for RT-qPCR or western blotting to measure METTL3 mRNA and protein levels, respectively. (B and C) METTL3 levels in lymph node, spleen, kidney, lung, tonsil, heart, and intestine from CSFV-infected or PBS-treated pigs were measured using IHC assay. 16×magnification (scale bar, 20 μm). These data are presented as the mean ± SD of data from three independent experiments. *p < 0.05; **p < 0.01; ***p < 0.001.
(TIF)

S2 Fig METTL3 has no effects on CSFV replication. (A and C) PK-15, IPEC-J2, and ST cells transfected with siMETTL3 and siCtrl (A) or pEGFP-METTL3 (0.2, 0.5 and 1 μg) (C) were infected with CSFV (MOI = 1). At 24 hpi, the total RNA were extracted or lysated and subjected to RT-qPCR or virus titration. (B and D) PK-15, IPEC-J2, and ST cells transfected with siMETTL3 or siCtrl (B) or pEGFP- METTL3 (0.2, 0.5 and 1 μg) (D) were infected with CSFV (MOI = 1). At 24 hpi, cells were harvested and subjected to western blotting using the indicated antibodies against GFP, METTL3, Npro, and β-actin or lysated for virus titration assay. These data are presented as the mean ± SD of data from three independent experiments. *p < 0.05, **p < 0.01, ***p < 0.001.
(TIF)

S3 Fig TLR4 regulates NF-κB pathway during CSFV infection. (A) PK-15 cells transfected with siTLR4 or siCtrl were infected with CSFV (MOI = 1), harvested at indicated time points and subjected to western blotting by using rabbit anti-TLR4/p-p65/p65/IKBα/Npro antibody along with β-actin as a loading control. (B-D) PK-15 cells transfected with siTLR4 or siCtrl were infected with CSFV (MOI = 1). The total mRNA in treated cells at indicated time points were extracted and subjected to RT-qPCR for detecting the mRNA levels of inflammatory cytokines. These data are presented as the mean ± SD of data from three independent experiments. **p < 0.01, ***p < 0.001.
(TIF)

S4 Fig METTL14 has no effect on IFN-I/III signaling pathway. (A) PK-15 cells transfected with siMETTL14 or siCtrl were infected with CSFV (MOI = 1) and then harvested for indicated time points for western blotting by using rabbit anti-p-TBK1, -TBK1, -p-IRF3, -IRF3, -p-STAT1, -STAT1, -Mx1 and Npro antibody, mouse anti-ISG15 antibody, and β-actin as a loading control. (B) PK-15 cells transfected with siMETTL14 or siCtrl were infected with CSFV (MOI = 1) and then harvested for indicated time points for RT-qPCR. These data are presented as the mean ± SD of data from three independent experiments. *p < 0.05, **p < 0.01, ***p < 0.001.
(TIF)

S1 Table siRNA duplxes used in this study. (DOCX)

S2 Table The primers used in this study. (DOCX)
