# Supplementary figures and images for: Classical swine fever virus non-structural protein 5B hijacks host METTL14-mediated m6A modification to counteract host antiviral immune response
Source: PLoS Pathog. 2024 Mar 29;20(3):e1012130. doi: 10.1371/journal.ppat.1012130 (PMC11006178; doi:10.1371/journal.ppat.1012130)

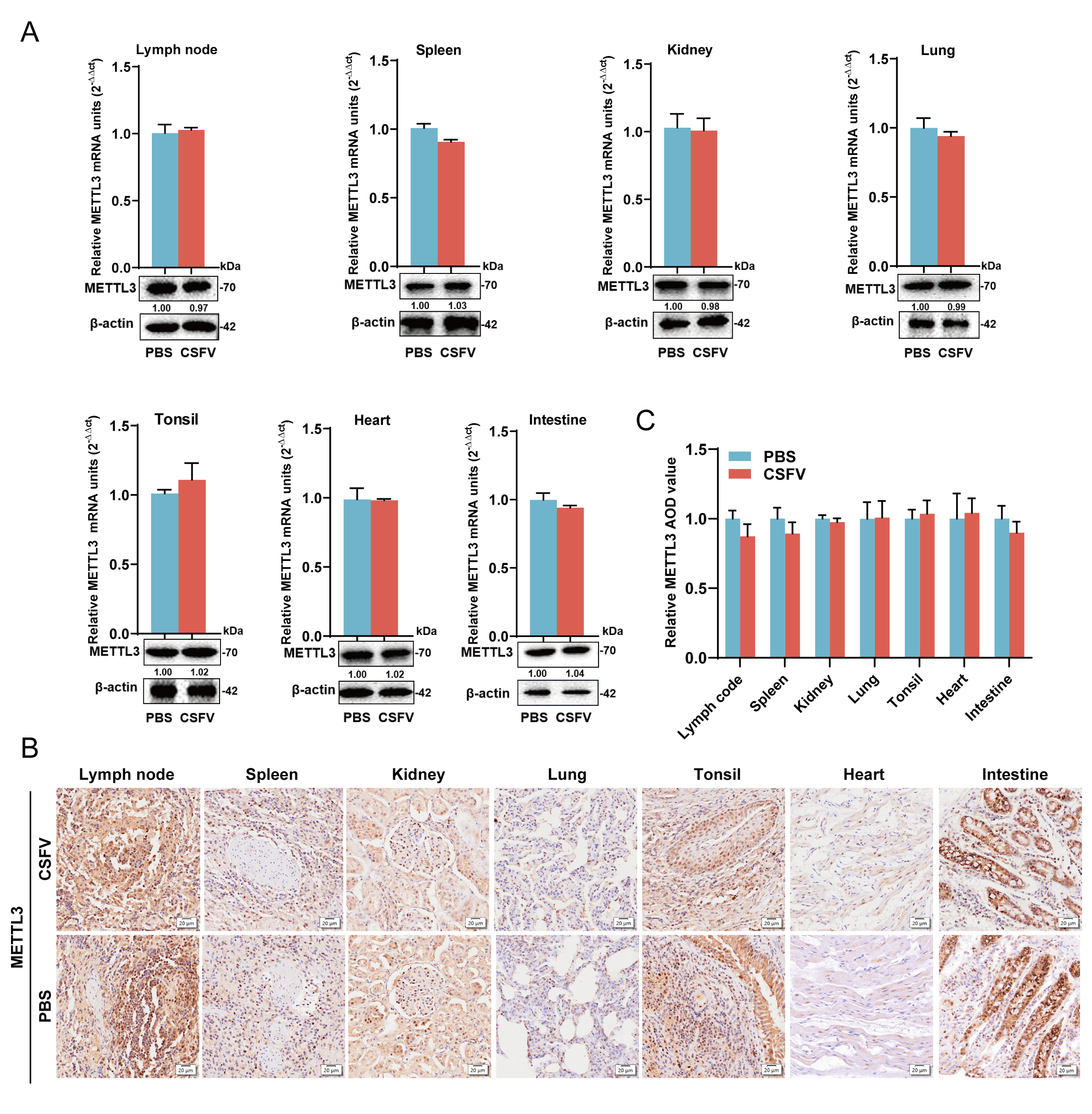

Supplement: S1 Fig — (A) Lymph node, spleen, kidney, lung, tonsil, heart, and intestine from CSFV-infected or PBS-treated pigs were extracted RNA or lysed for RT-qPCR or western blotting to measure METTL3 mRNA and protein levels, respectively. (B and C) METTL3 levels in lymph node, spleen, kidney, lung, tonsil, heart, and intestine from CSFV-infected or PBS-treated pigs were measured using IHC assay. 16×magnification (scale bar, 20 μm). These data are presented as the mean ± SD of data from three independent experiments. *p < 0.05; **p < 0.01; ***p < 0.001. (TIF) [file ppat.1012130.s001.tif]

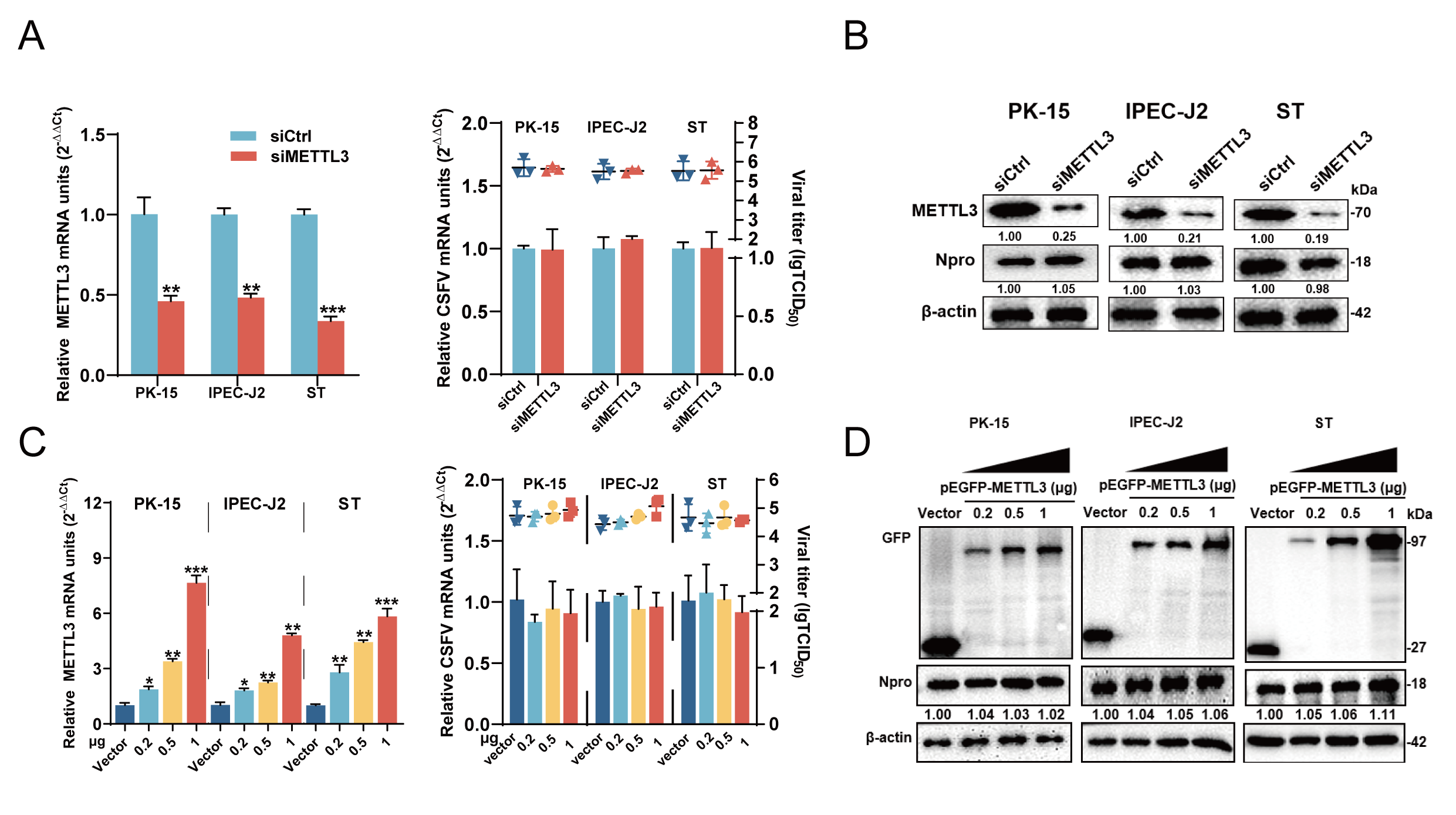

Supplement: S2 Fig — (A and C) PK-15, IPEC-J2, and ST cells transfected with siMETTL3 and siCtrl (A) or pEGFP-METTL3 (0.2, 0.5 and 1 μg) (C) were infected with CSFV (MOI = 1). At 24 hpi, the total RNA were extracted or lysated and subjected to RT-qPCR or virus titration. (B and D) PK-15, IPEC-J2, and ST cells transfected with siMETTL3 or siCtrl (B) or pEGFP- METTL3 (0.2, 0.5 and 1 μg) (D) were infected with CSFV (MOI = 1). At 24 hpi, cells were harvested and subjected to western blotting using the indicated antibodies against GFP, METTL3, Npro, and β-actin or lysated for virus titration assay. These data are presented as the mean ± SD of data from three independent experiments. *p < 0.05, **p < 0.01, ***p < 0.001. (TIF) [file ppat.1012130.s002.tif]

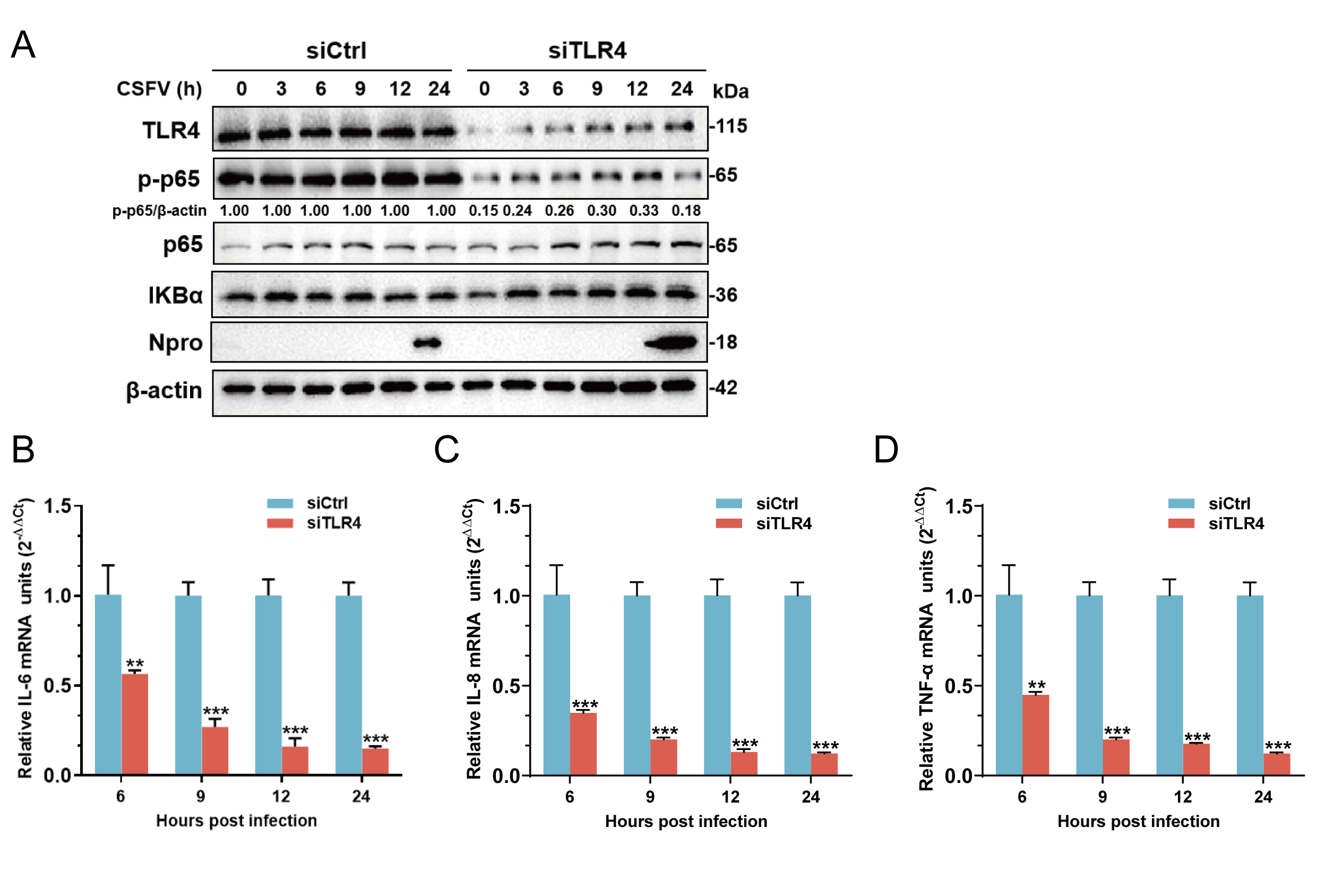

Supplement: S3 Fig — (A) PK-15 cells transfected with siTLR4 or siCtrl were infected with CSFV (MOI = 1), harvested at indicated time points and subjected to western blotting by using rabbit anti-TLR4/p-p65/p65/IKBα/Npro antibody along with β-actin as a loading control. (B-D) PK-15 cells transfected with siTLR4 or siCtrl were infected with CSFV (MOI = 1). The total mRNA in treated cells at indicated time points were extracted and subjected to RT-qPCR for detecting the mRNA levels of inflammatory cytokines. These data are presented as the mean ± SD of data from three independent experiments. **p < 0.01, ***p < 0.001. (TIF) [file ppat.1012130.s003.tif]

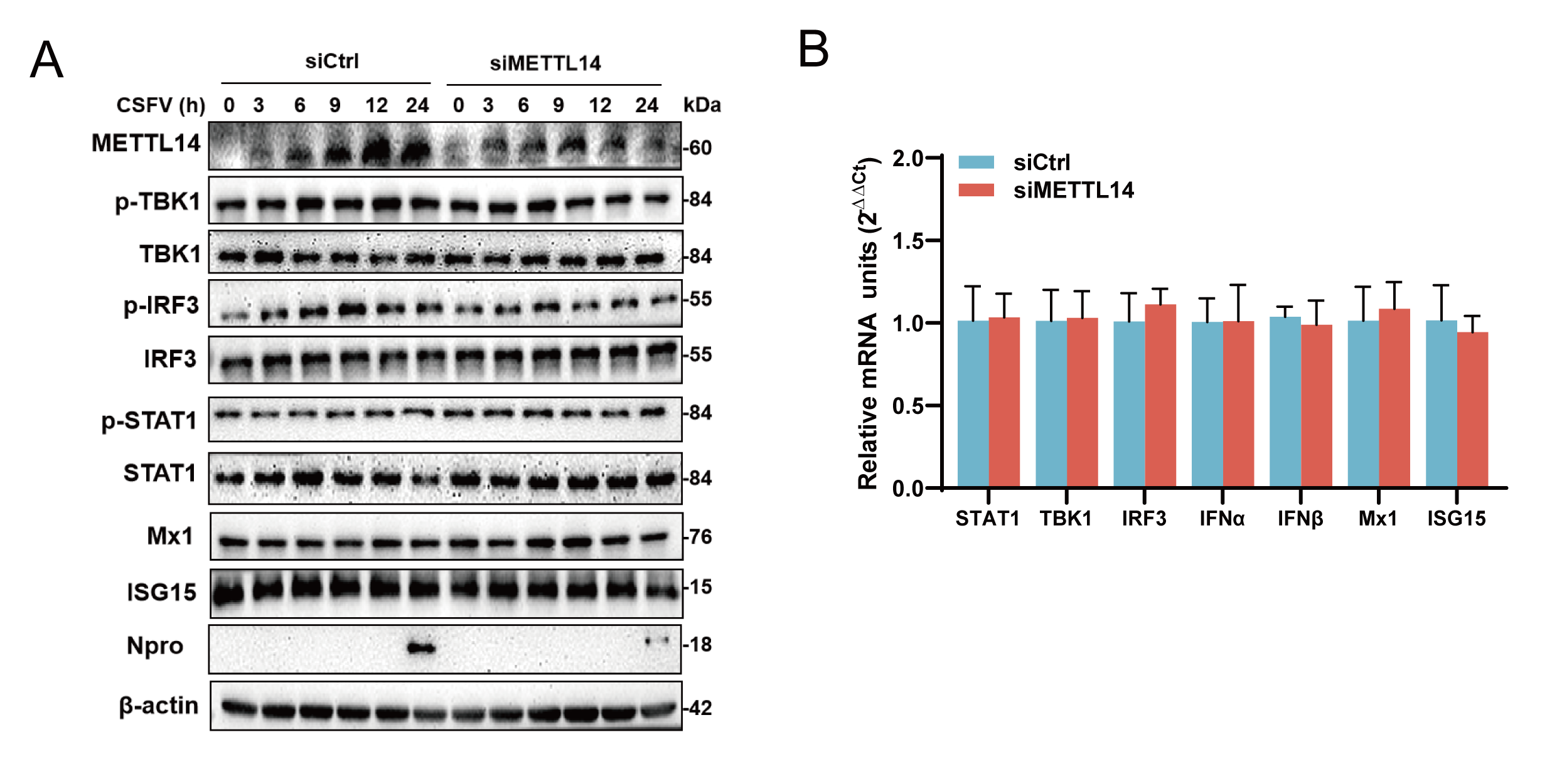

Supplement: S4 Fig — (A) PK-15 cells transfected with siMETTL14 or siCtrl were infected with CSFV (MOI = 1) and then harvested for indicated time points for western blotting by using rabbit anti-p-TBK1, -TBK1, -p-IRF3, -IRF3, -p-STAT1, -STAT1, -Mx1 and Npro antibody, mouse anti-ISG15 antibody, and β-actin as a loading control. (B) PK-15 cells transfected with siMETTL14 or siCtrl were infected with CSFV (MOI = 1) and then harvested for indicated time points for RT-qPCR. These data are presented as the mean ± SD of data from three independent experiments. *p < 0.05, **p < 0.01, ***p < 0.001. (TIF) [file ppat.1012130.s004.tif]
