# Supplementary material for: Classical swine fever virus non-structural protein 5B hijacks host METTL14-mediated m6A modification to counteract host antiviral immune response
Source: PLoS Pathog. 2024 Mar 29;20(3):e1012130. doi: 10.1371/journal.ppat.1012130 (PMC11006178; doi:10.1371/journal.ppat.1012130)
Supplement: S1 Table — (DOCX) [file ppat.1012130.s005.docx]

**S1 Table.**  **siRNA duplxes used in this study.**

| **Primer** | **Sequence (5'-3'）** | **Use** |
| --- | --- | --- |
| Negative Control | AATTCTCCGAACGTGTCACGT | Ctrl interference RNA |
| siYTHDF1 | CAGGCTGGAGAATAACGACAA | YTHDF1 interference RNA  YTHDF2 interference RNA |
| siYTHDF2 | GGACGUUCCCAAUAGCCAATT |  |
| siYTHDF3 | GGAUUAAAUCAGUAUCUAATT | YTHDF3 interference RNA |
| siMETTL3 | AGGAGCCAGCCAAGAAAUCAA | METTL3 interference RNA |
| siMETTL14 | TGGUGCCGUGUUAAAUAGCAA | METTL14 interference RNA |
