# Supplementary material for: Classical swine fever virus non-structural protein 5B hijacks host METTL14-mediated m6A modification to counteract host antiviral immune response
Source: PLoS Pathog. 2024 Mar 29;20(3):e1012130. doi: 10.1371/journal.ppat.1012130 (PMC11006178; doi:10.1371/journal.ppat.1012130)
Supplement: S2 Table — (DOCX) [file ppat.1012130.s006.docx]

**S2 Table. The primers used in this study.**

| **Primer** | **Sequence (5'-3'**） | **Gene ID** | **Use** |
| --- | --- | --- | --- |
| YTHDF1-F | GGACAAGTGGTTCTCAGGGG | 110257488 | RT-qPCR for detection of YTHDF1 |
| YTHDF1-R | CTGCCCATCAACAACTGTGC |  |  |
| YTHDF2-F | ACATTGTGGCTTCTAGCGGT | 100623159 | RT-qPCR for detection of YTHDF2 |
| YTHDF2-R | TGCTAGCAATGTCTGCCCAA |  |  |
| YTHDF3-F | CAAGTGCAGTCACAACAGCC | 106510070 | RT-qPCR for detection of YTHDF3 |
| YTHDF3-R | TTGTTCTGGTTGAAGCCGGT |  |  |
| FTO-F | TGCACTGATTGGTGGTGTGA | 100127165 | RT-qPCR for detection of FTO |
| FTO-R | GACCGGACGATTCTGGACTG |  |  |
| METTL3-F | CGTAGTGATAGTCCCGTGCC | 100513294 | RT-qPCR for detection of METTL3 |
| METTL3-R | GTTCAGGGGCCACAGATGTT |  |  |
| METTL14-F | GTGGTCGGGAAAGAAACCGA | 100525761 | RT-qPCR for detection of METTL14 |
| METTL14-R | GAGTAAAGCCGCCTCTGTGC |  |  |
| TLR4-F | TTCCGTGGCATTTTTGCTGG | 399541 | RT-qPCR for detection of TLR4 |
| TLR4-R | ATGCCCTCTGGGATACCTGT |  |  |
| TLR6-F | ACGTGGTAGAGAGTTGGCCT | 396621 | RT-qPCR for detection of TLR6 |
| TLR6-R | CCAGTGACTCCGATGGTGAC |  |  |
| MYD88-F | GCAGCATCCCTTGGATGTCA | 396646 | RT-qPCR for detection of MYD88 |
| MYD88-R | CTGCACAAACTGGGTATCGC |  |  |
| TRIF-F | CCCATTCCTCGTCACGATCTC | 100623776 | RT-qPCR for detection of TRIF |
| TRIF-R | TCAGACTGGTTTGGGATAGGTTT |  |  |
| CACTIN-F | CAGTCCGACTCTGGCGATG | 100521180 | RT-qPCR for detection of CACTIN |
| CACTIN-R | CTGGGGATGCAGAGCTAGAC |  |  |
| PIK3R1-F | ACAAGCGCATGAACAGCATT | 100623707 | RT-qPCR for detection of PIK3R1 |
| PIK3R1-R | GGCAAGTCCTCGTCGTCTTC |  |  |
| PTPN22-F | AGTGATGGACCAACAGAGCA | 100517760 | RT-qPCR for detection of PTPN22 |
| PTPN22-R | AGCAGGGTGCATAACTAGCC |  |  |
| AKT2-F | GGCTGCTTAAGAAGGACCCA | 100127478 | RT-qPCR for detection of AKT2 |
| AKT2-R | TCGTCGTCAAAGTACCGAGT |  |  |
| NFKBIL1-F | TCGAAGATGATGCTTCCCAC | 100154350 | RT-qPCR for detection of NFKBIL1 |
| NFKBIL1-R | GGCTCGCTCTCGGAACAG |  |  |
| RAB11FIP2-F | CTTTTGGGTCCTCAGCGACT | 100153733 | RT-qPCR for detection of RAB11FIP2 |
| RAB11FIP2-R | GATGAGTTTGAGCGATGGCG |  |  |
| GRAMD4-F | CGCTTTGCTGGAAAAGCACT | 110260607 | RT-qPCR for detection of GRAMD4 |
| GRAMD4-R | ATTTCCTGTGGCAGTCACCC |  |  |
| IL-6-F | AGGGAAATGTCGAGGCTGTG | 399500 | RT-qPCR for detection of IL-6 |
| IL-6-R | TCCACTCGTTCTGTGACTGC |  |  |
| IL-8-F | AGCCCGTGTCAACATGACTTCC | 396880 | RT-qPCR for detection of IL-8 |
| IL-8-R | GAAGTTGTGTTGGCATCTTTACTGA |  |  |
| TNF-α-F | GGCAGAGTGGGTATGCCAAT | 397086 | RT-qPCR for detection of TNF-α |
| TNF-α-R | GAGGTACAGCCCATCTGTCG |  |  |
| CSFV-F | GAACTGGGCTAGCCATG | Accession: AF092448 | RT-qPCR for detection of CSFV |
| CSFV-R | ACTGTCCTGTACTCAGGAC |  |  |
| GAPDH-F | GAAGGTCGGAGTGAACGGATTT | 396823 | RT-qPCR for detection of GAPDH |
| GAPDH-R | TGGGTGGAATCATACTGGAACA |  |  |
